# Spatial isomerism regulates host–guest distance for highly efficient organic room-temperature phosphorescence

**DOI:** 10.1039/d6sc04960e

**Published:** 2026-08-03

**Authors:** Hao Sun, Chen Lu, Jianye Gong, Nuan Wen, Ru Pang, Jin Tao, Xiaolang Xiong, Guoyu Jiang, Ben Zhong Tang, Jianguo Wang

**Affiliations:** a College of Chemistry and Chemical Engineering, Institute of Green Chemistry and Environment, Institutes of Biomedical Sciences, Inner Mongolia Key Laboratory of Synthesis and Application of Organic Functional Molecules, Inner Mongolia University Hohhot 010021 P. R. China wangjg@iccas.ac.cn jiangguoyu@mail.ipc.ac.cn; b Guangdong Basic Research Center of Excellence for Aggregate Science, School of Science and Engineering, The Chinese University of Hong Kong (Shenzhen) Longgang Shenzhen Guangdong 518172 P. R. China tangbenz@cuhk.edu.cn

## Abstract

The host–guest organic room-temperature phosphorescent (RTP) materials based on Dexter triplet–triplet energy transfer (TTET) have demonstrated significant application potential in fields such as anti-counterfeiting, information encryption, and stimulus-responsive sensing. Nevertheless, most reported RTP materials have focused on tuning host–guest energy-level alignment. Strategies capable of structurally manipulating host–guest distances at the molecular level remain exceedingly rare. Herein, a spatial isomerism strategy is employed that precisely modulates the distance between host and guest molecules, thereby significantly increasing the phosphorescence quantum yield from 0.81% to 8.97% and extending the lifetime from 23 to 158 ms through enhanced TTET. Among the doped systems, the crystal structure and electronic properties of the guests indicate that the degree of distortion and electrostatic complementarity determine the phosphorescence quantum yield. Further quantum mechanics/molecular mechanics calculations quantitatively reveal that the host–guest average distance can be compressed from 3.36 Å to 2.86 Å, thereby enhancing the distance-sensitive TTET process and achieving a simultaneous increase of more than one order of magnitude in phosphorescence performance. In addition, all the doped systems exhibit significant acid–base stimulus-responsiveness, enabling reversible control of phosphorescent color. This tunability provides the possibility for constructing multi-level environment-responsive anti-counterfeiting and information encryption platforms.

## Abstract 2

Spatial isomerism compresses host–guest distance from 3.36 to 2.86 Å by balancing guest conformation with electrostatic complementarity, enhancing Dexter TTET and increasing *Φ*_Phos._ from 0.81% to 8.97% and *τ*_P_ from 23 to 158 ms.

## Graphical abstract

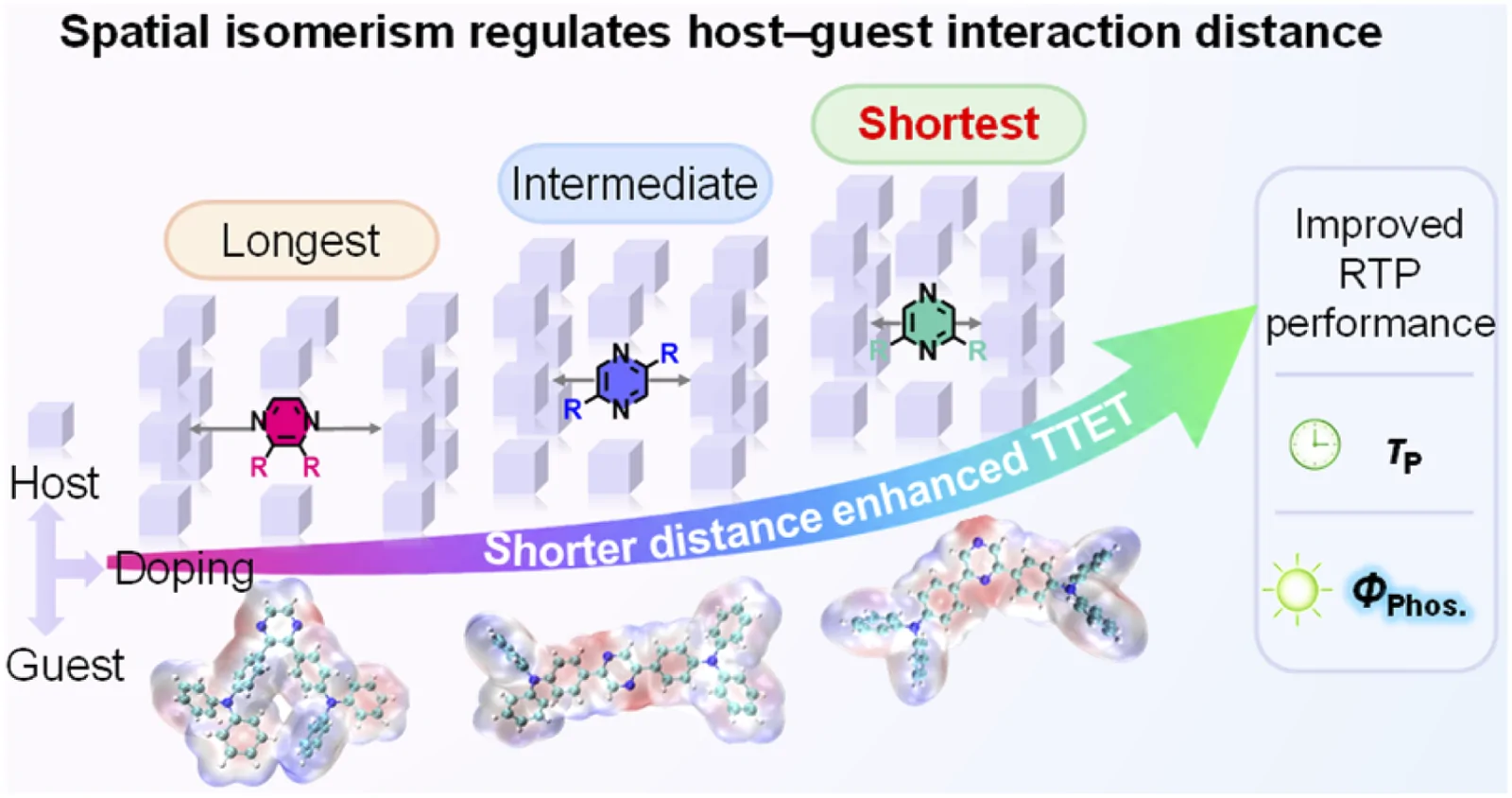

## Introduction

Organic room-temperature phosphorescent (RTP) materials have attracted increasing attention for applications in information encryption, bioimaging and intelligent sensing, owing to their unique ability to stabilize long-lived triplet excitons without relying on heavy-metal components.^1–6^ Nevertheless, the development of efficient purely organic RTP systems remains a formidable challenge, primarily due to inherently large nonradiative decay rates and weak spin–orbit coupling (SOC) constants in organic compounds.^7–10^ Over the past decade, considerable progress has been achieved through molecular design strategies that promote intersystem crossing (ISC), including the incorporation of aromatic carbonyl groups to enhance n–π* transitions and the introduction of heavy atoms to strengthen SOC.^11–15^ Despite these advances, realizing high RTP efficiency in purely organic systems remains intrinsically challenging, as triplet excitons are prone to dissipation *via* nonradiative pathways such as intramolecular vibration and rotational relaxation.^16–20^ To suppress these channels, various solid-state confinement strategies have been developed, including crystal engineering,^21,22^ H-aggregation,^23,24^ polymer encapsulation^25–28^ and host–guest doping.^29,30^ Among them, host–guest doping systems are particularly attractive because the guest structure can be flexibly designed, while the host matrix provides effective confinement. Their cooperative effects enable the regulation of phosphorescence colour, efficiency, lifetime, and stability.^31–33^ Importantly, the ordered crystal lattice of the host provides an ideal platform for probing intermolecular energy transfer processes at the molecular level, offering opportunities to elucidate fundamental photophysical mechanisms underlying organic RTP.^34–36^

In host–guest RTP systems, excellent phosphorescence performance critically depends on the effective harvesting and migration of triplet excitons. These processes are generally mediated by Förster resonance energy transfer (FRET) and Dexter-type triplet–triplet energy transfer (TTET). FRET operates through long-range (1–10 nm) dipole–dipole interactions and predominantly involves singlet-to-singlet transitions.^37^ Triplet-to-singlet FRET (TS-FRET) becomes viable only when SOC is sufficiently strong.^38^ In contrast, TTET proceeds *via* short-range (<1 nm) electron exchange, requiring direct orbital overlap or van der Waals contact between triplet donor and triplet acceptor.^39–41^ Importantly, TTET bypasses the necessity for additional ISC in the acceptor and minimizes exciton loss during spin conversion, making it inherently more favorable for achieving efficient organic RTP.^42^ According to the Dexter equation *k*_TTET_ = *KJ* exp(−2*R*/*L*), TTET rate constant decreases exponentially with increasing intermolecular distance (*R*),^43,44^ underscoring the decisive role of precise molecular spacing in enabling efficient triplet energy transfer.^45–47^ To date, most studies have focused on engineering host–guest energy-level alignment to promote forward triplet transfer or suppress reverse transfer.^48,49^ In sharp contrast, strategies capable of structurally manipulating host–guest distances at the molecular level remain exceedingly rare. A major obstacle lies in the intrinsically low dopant concentrations typically employed in host–guest crystals, which render guest positions nearly invisible to conventional crystallographic analysis.^50,51^ As a result, rational design and precise control of host–guest proximity remain largely inaccessible. Although TTET is widely recognized as a key mechanism for organic RTP, the field still lacks a generalizable approach to directly manipulate intermolecular spacing within doped crystals, representing a fundamental bottleneck in the development of high-performance host–guest RTP materials.

Herein, we address this challenge by proposing a spatial isomerism-guided strategy that enables precise regulation of host–guest intermolecular distances, thereby promoting TTET efficiency in organic RTP materials (Scheme 1). Three spatial isomers of triphenylamine (TPA)-modified pyrazine (OBQ, MBQ, and PBQ),^52^ identical in molecular formula yet distinct in steric configuration and dipole distribution, were employed as guest emitters within a benzophenone (BP) host matrix. Subtle differences in molecular geometry and electrostatic properties lead to markedly different embedding modes in the host lattice, resulting in pronounced variations in host–guest separation. Steady-state and delayed emission spectra measurements reveal a progressive quenching of BP phosphorescence accompanied by a synchronous enhancement of guest emission with increasing dopant concentration, confirming an efficient TTET process. Among the three isomers, MBQ exhibits the most favorable host–guest compatibility through a suitable balance between molecular conformation and electrostatic characteristics. Its suitable molecular conformation, relatively large dipole moment (2.31 D), and ΔESP value (56.88 kcal mol^−1^) close to those of BP collectively favor compact accommodation within the BP lattice and further strengthen the host–guest interactions. Powder X-ray diffraction (PXRD) analysis confirms that none of the dopants perturb the host's crystal framework, indicating that the observed differences in RTP performance primarily originate from variations in local intermolecular geometry rather than lattice distortion. This interpretation is further corroborated by quantum mechanics/molecular mechanics (QM/MM) calculations, which reveal that BP/MBQ achieves the shortest host–guest average distance (*x̄*(C_host_–H_guest_) = 2.86 Å), compared with BP/PBQ (*x̄*(C_host_–H_guest_) = 3.26 Å) and BP/OBQ (*x̄*(C_host_–H_guest_) = 3.36 Å), directly correlating with its superior phosphorescence performance (*Φ*_Phos._ = 8.97%, *τ*_P_ = 158 ms). Beyond enhanced RTP performance, all doped systems exhibit pronounced acid/base responsive behavior, enabling reversible modulation of phosphorescence color. This responsiveness allows the construction of advanced stimulus-response architectures, providing high design flexibility for multi-level, environment-adaptive anti-counterfeiting and information encryption applications. Collectively, these findings demonstrate that molecular spacing precisely regulated through spatial isomerism represents a decisive structural parameter governing TTET. This work thus establishes a generalizable structural design principle for the development of next-generation high-performance organic RTP materials.

**Scheme 1:**
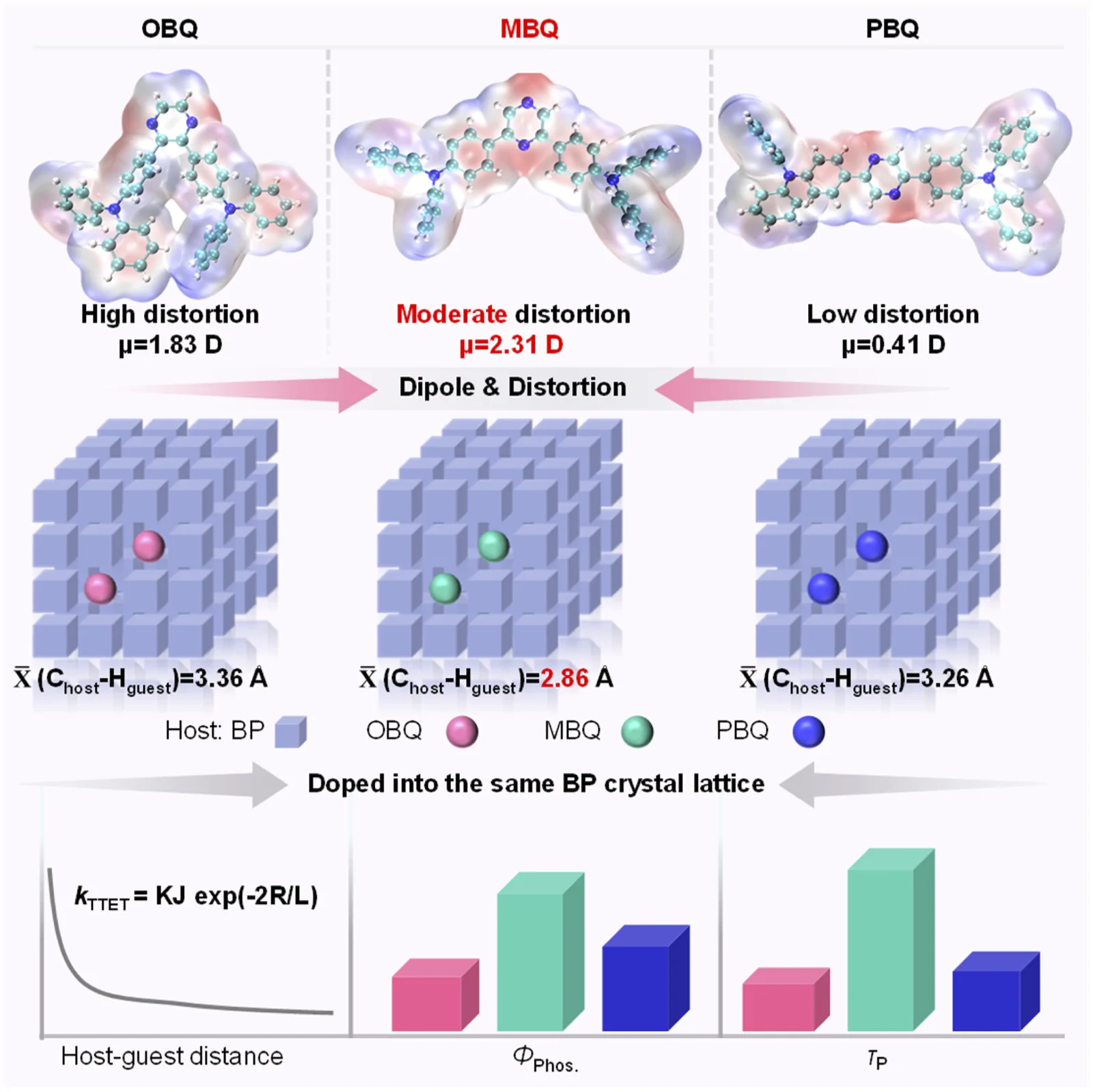
Spatial isomerism strategy precisely regulates host–guest distance to improve TTET efficiency and RTP performance. *k*_TTET_ = *KJ* exp(−2*R*/*L*). *K* is a prefactor related to specific orbital interactions between the host and the guest. *J* is the spectral overlap integral. *R* is the host–guest distance in the Dexter model. *L* is the sum of the van der Waals radii of the host and guest.^43^*x̄*(C_host_–H_guest_) is host–guest average C_host_–H_guest_ distance. *Φ*_Phos._: phosphorescence quantum yield of doped materials. *τ*_P_ is phosphorescence lifetime.

## Results and discussion

Based on the design rationale outlined above, BP was selected as the host matrix due to its high triplet energy level and efficient ISC capability, both essential for serving as a triplet exciton donor in host–guest RTP materials.^53^ To exploit spatial isomerism as a molecular handle for regulating host–guest distance, a series of triphenylamine-modified pyrazine derivatives with different substitution positions were designed as guest emitters. Pyrazine was chosen as the rigid planar scaffold compatible with multi-site substitution, while TPA introduces steric differentiation and modulates host–guest interactions.^54^ The propeller-like geometry of TPA and its electron-donating character result in distinct steric profiles and dipole distributions among the spatial isomers, while preserving identical molecular formulas.^55^ As a consequence, these isomers adopt different embedding modes within the BP crystal framework, enabling modulation of host–guest intermolecular distances without perturbing the overall crystal packing of the host (Fig. 1a). All guest molecules were synthesized *via* a one-step Suzuki coupling reaction, and their structures and purities were confirmed by NMR spectroscopy, mass spectrometry, and high-performance liquid chromatography (HPLC) (Fig. S1–S13). Leveraging the low melting point of BP (48 °C), host–guest doped solids were conveniently prepared *via* melt-casting. BP and guest compounds were mixed at various mass ratios (200 : 1–3000 : 1), heated to 60 °C to ensure homogeneous melting, and cooled to room temperature to induce crystallization. The low-temperature and solvent-free melt-casting process is suitable for batch preparation. The molten materials can also be directly cast into molds or onto substrates, showing potential compatibility with patterned device fabrication. To quantitatively assess the mass ratio dependence of RTP performance, the absolute photoluminescence quantum yields, phosphorescence quantum yields, and lifetimes of BP/OBQ, BP/MBQ, and BP/PBQ were measured over a range of mass ratios. At a mass ratio of 1000 : 1, the three systems exhibited optimal phosphorescence quantum yield or lifetime (Fig. S14 and Table S1). This optimized ratio was applied to the preparation of BP/OBQ, BP/MBQ, and BP/PBQ in all subsequent experiments.

**Fig. 1:**
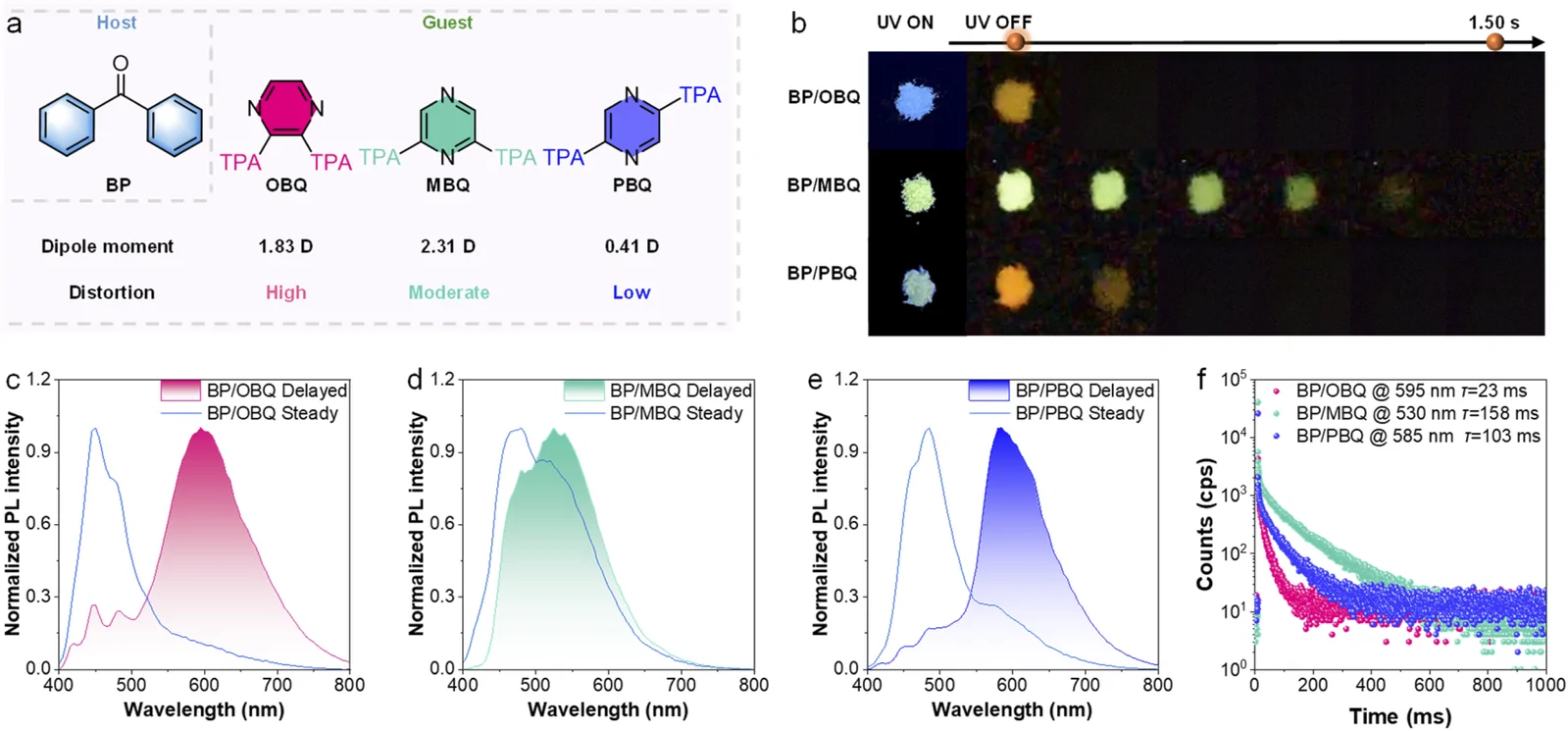
(a) Molecular structures of the host and guest molecules, as well as the dipole moments of the guest molecules. Distortion represents the degree of distortion of guest molecules. (b) Photographs of host–guest doped materials (mass ratio 1000 : 1) under ambient conditions before and after turning off the 365 nm UV. The samples were irradiated with a 365 nm LED at a power density of 80 mW cm^−2^ for 15 s. Steady-state and delayed emission spectra of BP/OBQ (c), BP/MBQ (d), and BP/PBQ (e). (f) Time-resolved emission decay curves of three host–guest doped materials. Excitation wavelength: *λ*_ex_: 360 nm. Delay time 1 ms.

To investigate how spatial isomerism influences triplet-state dynamics and RTP performance, the photophysical properties of the three host–guest systems were systematically compared. Upon removal of UV excitation, the three doped systems exhibit distinct afterglow behaviors (Fig. 1b), reflecting isomer-dependent triplet relaxation. Among the doped systems, BP/MBQ showed the greatest afterglow contribution in the steady-state emission spectra, followed by BP/PBQ and BP/OBQ. This trend suggests that BP/MBQ possesses the most favorable phosphorescence performance, while BP/OBQ exhibits the weakest, in agreement with the phosphorescence images (Fig. 1c–e and Table S1). Specifically, BP/OBQ displays weak phosphorescence centered at 595 nm, with a short lifetime (*τ*_P_ = 23 ms) and a very low *Φ*_Phos._ of 0.81%, consistent with its faint, rapidly decaying afterglow. BP/PBQ exhibits a similar phosphorescence maximum at 585 nm, but with a longer lifetime (*τ*_P_ = 103 ms) and moderate *Φ*_Phos._ of 1.44%. Notably, BP/MBQ shows intense delayed green emission with the highest *Φ*_Phos._ of 8.97%, featuring a long lifetime of 158 ms (Fig. S15). These results demonstrate that minor differences in substitution position profoundly influence triplet exciton dynamics, leading to distinct RTP intensity and decay behaviors. This comparative isomeric platform establishes a relationship between structure and properties, directly revealing the connection between subtle molecular differences and RTP behavior, including emission color, intensity, and lifetime.

To clarify the origin of the observed RTP, the delayed emission spectra of the isolated guest molecules in dilute solutions at 77 K were compared with those of the corresponding host–guest systems at room temperature (Fig. S16). At 77 K, OBQ and MBQ exhibit phosphorescence bands centered at approximately 575 and 530 nm, respectively, while PBQ shows no detectable phosphorescence. For OBQ, the low-temperature phosphorescence maximum at 575 nm closely resembles the RTP maximum of the BP/OBQ doped system at 595 nm. Meanwhile, the characteristic phosphorescent bands of the BP host at 420, 445, and 480 nm remain clearly discernible in the delayed emission spectra of BP/OBQ, indicating that the host and guest triplet emissions can be spectrally distinguished. These results demonstrate that the RTP emission centered at approximately 595 nm primarily originates from the triplet excited state of the OBQ guest rather than the intrinsic phosphorescence of the host matrix. In the case of PBQ, although no intrinsic phosphorescence was detected for the guest alone, the BP/PBQ shows distinct host phosphorescence peaks at 420 nm, 445 nm, and 480 nm, with an additional emission at 585 nm that is well separated from the host emission, suggesting that RTP in BP/PBQ primarily arises from guest triplet states (Fig. 1e). Notably, while MBQ exhibits low-temperature phosphorescence at 500 nm and 530 nm (Fig. S16b), the BP/MBQ doped system shows an additional shoulder peak at 480 nm. To clarify the origin of this feature, MBQ was incorporated into poly(methyl methacrylate) (PMMA), and the delayed emission spectra of PMMA@MBQ were measured under vacuum from 157 K to 323 K. The peak near 460 nm gradually intensifies upon heating, accompanied by a simultaneous increase in the emission lifetime. The spectral position of this emission is similar to the shoulder peak near 480 nm in BP/MBQ (Fig. S17). These results indicate that the shoulder peak at 480 nm in BP/MBQ arises from delayed fluorescence, while the peak at 530 nm originates from the guest triplet state. Temperature-dependent delayed emission measurements of the doped systems from 77 K to 297 K (Fig. S18–S20) reveal that both phosphorescence intensity and lifetime decrease significantly with increasing temperature, consistent with enhanced nonradiative deactivation of triplet excitons. This temperature dependence confirms that RTP in these host–guest systems primarily originates from guest triplet states rather than thermally activated processes. Collectively, these observations establish that RTP emission in all three doped systems is dominated by guest radiative decay, while spatial isomerism modulates triplet-state dynamics and thus governs phosphorescence intensity and lifetime. Such an emission origin suggests that the triplet excited states of the guest molecules are efficiently populated in the BP matrix. Considering the efficient ISC of BP and its ability to generate abundant triplet excitons, we thus assumed that in these host–guest systems, BP serves as a triplet exciton reservoir, transferring triplet energy to the guest molecules and thereby sensitizing guest phosphorescence through a TTET process.

To verify the TTET process between the host and guest, a series of spectroscopic and theoretical analyses were carried out. Taking BP/MBQ as a representative system, its significantly improved photophysical properties are shown in Fig. 2, while results for other doped materials are provided in Fig. S21–S25. First, solid-state absorption spectra were measured to examine ground-state interactions between host and guest. The absorption of the host–guest doped system can be thought of as the superposition of the absorption of the host and guest, with no new absorption bands observed (Fig. 2a and S21). This indicates that the host and guest molecules coexist as a physical mixture upon doping, without forming a ground-state charge-transfer complex. Second, to identify the energy donor in the doped system, delayed emission spectra and time-resolved emission decay curves were collected under different excitation wavelengths. As shown in Fig. 2b and S22, compared to excitation at 360 nm, the spectral intensity and the lifetime decreases drastically under 430 nm excitation. Based on the absorption spectra profiles of BP and MBQ, the excitation wavelengths of 360 and 430 nm selectively excite BP or not, respectively. This result indicates that BP serves as the primary triplet donor for the delayed emission of the doped system. Similar excitation wavelength-dependent behavior was also observed for the BP/OBQ and BP/PBQ systems (Fig. S23). To further investigate the energy transfer process, the delayed emission spectra and time-resolved emission decay curves were measured as a function of host–guest ratios. As the guest content increased, the intrinsic phosphorescence of BP at 420 nm was progressively quenched, both in intensity (Fig. 2c) and lifetime (Fig. 2d). In contrast, the guest phosphorescence peak at 530 nm is gradually enhanced, and its phosphorescence lifetime increases from 29 to 106 ms (Fig. 2e). Similar reciprocal changes were observed for BP/OBQ (Fig. S24) and BP/PBQ (Fig. S25). These results support that the growing phosphorescence contribution of the guest comes at the expense of BP's triplet excited state.

**Fig. 2:**
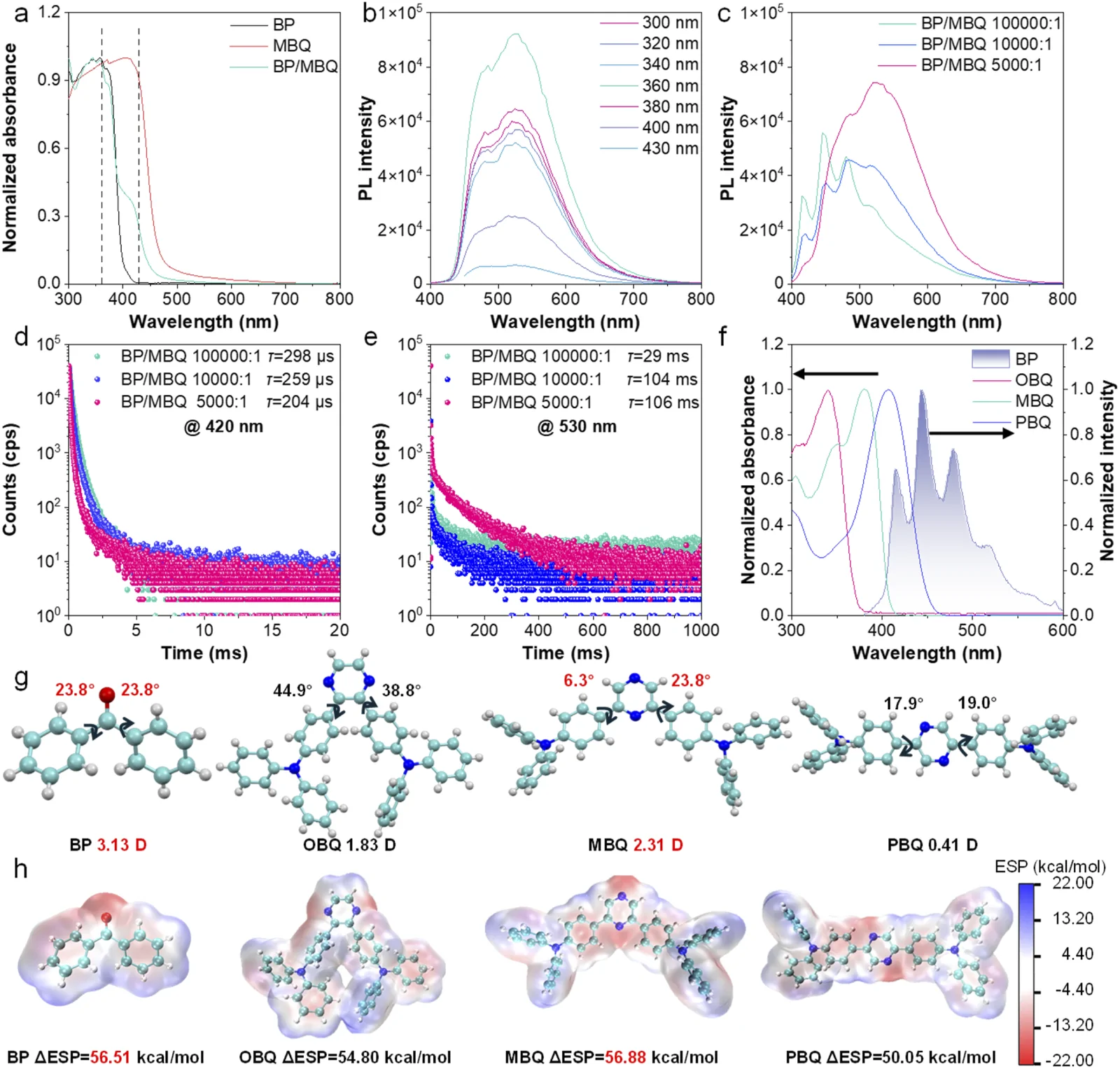
(a) Normalized UV-vis absorption spectra of solid BP, MBQ and BP/MBQ. The dashed lines are the selected excitation wavelengths at 360 and 430 nm. (b) Delayed emission spectra of BP/MBQ under different excitation wavelengths. (c) Delayed emission spectra of BP/MBQ under different mass ratios. (d) Time-resolved emission decay curves of BP/MBQ at 420 nm. (e) Time-resolved emission decay curves of BP/MBQ at 530 nm. (f) Normalized delayed emission spectra of BP solids (delay time 1 ms) and UV-vis absorption spectra of guests in *n*-hexane solution (1.0 × 10^−6^ M). (g) Molecular conformations, dihedral angles, and dipole moments of the host and guest molecules in their single crystal structures. (h) Electrostatic potential distributions and electrostatic potentials difference of the host and guest molecules, respectively. ΔESP is the difference between the maximum and minimum ESP values (the molecular geometries were extracted from the corresponding single crystals and calculated based on B3LYP/6-311G* basis set and processed using Multiwfn and VMD).

To exclude a dominant contribution from TS-FRET, the delayed emission spectra of BP and the absorption spectra of the guests were compared. As shown in Fig. 2f, PBQ exhibits the largest spectral overlap with BP, whereas BP/PBQ shows a lower phosphorescence quantum yield and a shorter lifetime than BP/MBQ. This result indicates that TS-FRET is not the primary factor governing the observed RTP. In addition, to evaluate the intrinsic ISC capability of the guests, the excited-state energy levels, SOC matrix elements, and natural transition orbitals (NTOs) of the guests were calculated. The NTO analyses reveal that, the ISC process from the (π, π*) S_1_ state to the (π, π*) T_*n*_ state is unfavorable for OBQ and PBQ (Fig. S26–S29), according to El-Sayed's rule. In MBQ, the transitions from the (π, π*) S_1_ state to the (n, π*) T_*n*_ state are more favorable for ISC. Nevertheless, the small SOC values (0.58 and 0.39 cm^−1^) indicate that this process remains weak. Collectively, these results demonstrate that TS-FRET can only make a limited contribution to the observed phosphorescence. Therefore, these results strongly support that the observed RTP is predominantly governed by a TTET process from the triplet state of BP to the guest triplet manifold.

Having established the origin of phosphorescence and the TTET pathway, the effect of host–guest distance on phosphorescent performance was investigated. The crystal structure of BP (CCDC: 245188) reveals a dihedral angle of 23.8° between the carbonyl group and the adjacent benzene ring, exhibiting moderate molecular distortion (Fig. 2g and Table S2). Given the extremely low doping concentration, the guest molecules are presumed to exist as isolated entities within the BP host, rather than forming aggregates. Consequently, variations in phosphorescence can be directly attributed to the intrinsic structure and electronic properties of the guest molecules, and their spatial arrangement relative to the host. Notably, the three guest isomers exhibit significant differences in molecular planarity and conformational distortion, which critically determine their embedding geometry and intermolecular distances within the BP lattice, thereby influencing TTET efficiency. Specifically, OBQ (CCDC: 2545022) adopts a highly distorted conformation, with pyrazine-TPA dihedral angles of 44.9°/38.8° and torsion angles of 46.0°/38.2°, indicating the strongest spatial non-planarity. MBQ (CCDC: 2545024) shows moderate distortion, with dihedral angles of 6.3°/23.8° and torsion angles of 11.5°/28.4°. PBQ (CCDC: 2545026) exhibits the highest planarity, with dihedral angles of 17.9°/19.0° and torsion angles of 19.3°/17.8° (Fig. S30). A larger dipole moment enhances intermolecular electrostatic interactions by creating more pronounced positive and negative surface potential zones. BP has a dipole moment of 3.13 D. Among the guest, MBQ possesses the largest dipole moment (2.31 D), indicating the strongest dipole–dipole interaction with BP. In contrast, OBQ and PBQ have smaller dipole moments (1.83 D and 0.41 D, respectively) due to their higher molecular symmetry. Surface electrostatic potential calculations further reveal that the ΔESP value of MBQ (56.88 kcal mol^−1^) closely matches that of BP (56.51 kcal mol^−1^), whereas OBQ and PBQ show poorer complementarity (54.80 and 50.05 kcal mol^−1^, respectively, Fig. 2h). Nevertheless, the observed RTP trend cannot be attributed to dipole–dipole interactions, molecular shape, or planarity alone; rather, it arises from the synergistic interplay of all three factors. OBQ has a larger dipole moment than PBQ but exhibits poorer RTP performance, whereas the higher planarity of PBQ does not lead to the best performance. These contrasting results indicate that electrostatic interactions and molecular conformation act cooperatively in regulating host–guest compatibility. Among the three guests, MBQ combines moderate molecular distortion with the largest dipole moment and a ΔESP value close to that of BP, resulting in the most favorable overall compatibility with the BP host. This synergy between structural and electronic properties directly enhances TTET efficiency, resulting in the significantly improved phosphorescence quantum yield and lifetime observed in the BP/MBQ system. Collectively, these findings provide compelling molecular-scale evidence that host–guest distance is a decisive factor governing TTET efficiency and RTP performance in purely organic systems.

The local microenvironment, including molecular conformation, intermolecular distance and noncovalent interactions, plays a critical role in determining the photophysical properties of host–guest systems. Given the extremely low content of guest molecules (<0.1%), formation of host–guest eutectic compounds is challenging, rendering traditional characterization techniques (*e.g.*, X-ray single crystal diffraction, scanning electron microscopy, and transmission electron microscopy) insufficient to resolve the precise molecular conformation of the guests within the host matrix.^56–58^ PXRD patterns of the pristine BP host and the doped system show complete overlap in peak positions, confirming that all doped samples retain the same crystal packing (Fig. S31). This indicates that observed differences in RTP performance primarily arise from variations in guest geometry rather than host lattice distortion, providing a solid foundation for subsequent QM/MM calculations.

To quantitatively analyze host–guest distance and weak noncovalent interactions, QM/MM simulations were performed to model the conformational behavior of guest molecules embedded in the BP host lattice (additional details are provided in the SI).^59^ The optimized ground state structures are shown in Fig. S32. To evaluate how host–guest separation influences phosphorescence, the guest molecules and their surrounding host molecules were extracted for analysis. Hirshfeld surface analysis (Fig. 3a) was conducted using Multiwfn and visualized in VMD to assess the strength of interactions. Quantitative analysis using pie charts (Fig. 3b) and fingerprint plots (Fig. S33–S35) indicates that C–C and C–H interactions account for over 85% of weak interactions in the doped systems, with other interaction types contributing less than 15%. C–C interactions promote π–π stacking, potentially quenching fluorescence, whereas C–H interactions spatially constrain guest molecules, reducing nonradiative decay and enhancing phosphorescence intensity. These analyses indicate that the extracted clusters preserve the principal host–guest interaction environment, providing a reliable structural basis for the subsequent quantitative analysis of host–guest separation.

**Fig. 3:**
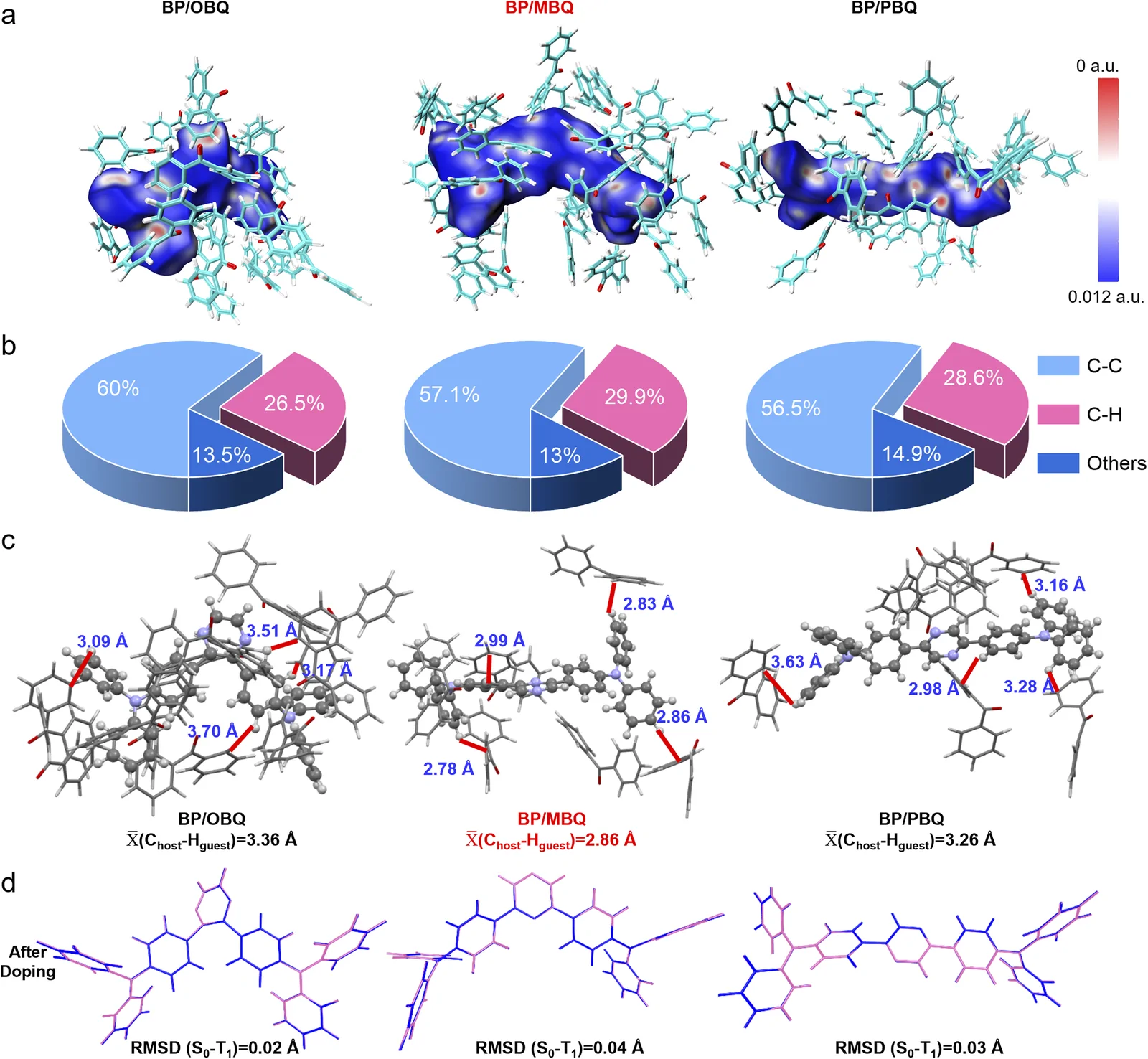
(a) QM/MM simulates the 3D Hirshfeld surfaces for BP/OBQ, BP/MBQ and BP/PBQ doped systems. The interactions are shown in red on the surface. (b) Pie charts of the proportions of intermolecular C–C, C–H, and other interactions relative to the total intermolecular interactions in BP/OBQ, BP/MBQ and BP/PBQ, calculated from QM/MM simulation structures. (c) QM/MM simulates the *x̄*(C_host_–H_guest_) between a guest molecule and the surrounding host molecules. (d) Overlaid molecular geometries of OBQ, MBQ and PBQ after doping into BP, shown for the S_0_ (blue) and T_1_ (pink) states.

In solid-state systems, short-range C–H contacts at the molecular periphery often constitute the most directional interactions relevant to TTET, accurately reflecting the spatial scale of energy transfer.^50,60^ Given the exponential dependence of TTET on intermolecular distance, an effective metric for host–guest proximity is necessary. We introduced the host–guest average C–H distance (*x̄*(C_host_–H_guest_)) to quantify the spatial separation within the host–guest cluster model. The results show that BP/MBQ, which combines moderate molecular distortion with the largest dipole moment, exhibits the shortest *x̄*(C_host_–H_guest_) (2.86 Å) and the highest C_host_–H_guest_ interaction ratio (29.9%). In BP/PBQ, the highly planar and symmetric guest leads to weaker dipole–dipole interactions, increasing the *x̄*(C_host_–H_guest_) to 3.26 Å with a C_host_–H_guest_ ratio of 28.6%. BP/OBQ, despite having a dipole moment of 1.83 D, is severely distorted, limiting close packing and resulting in the lowest C_host_–H_guest_ ratio (26.5%) and the largest *x̄*(C_host_–H_guest_) (3.36 Å, Fig. 3c). These results demonstrate that neither dipole moment nor molecular distortion alone is sufficient to minimize host–guest separation, an optimal balance of both factors is required. This distance-regulating effect, governed by spatial isomerism, provides direct structural evidence for enhanced TTET in BP/MBQ, rationalizing its superior RTP performance.

To further demonstrate the stability of host–guest packing upon excitation, root-mean-square deviation (RMSD) values of the guest molecules in the excited state were analyzed before and after doping. In the triplet state, the isolated OBQ, MBQ, and PBQ molecules exhibit relatively large structural deviations, with RMSD values ranging from 0.79 to 1.05 Å (Fig. S36). After doping into the BP, the corresponding RMSD values are markedly reduced to 0.02–0.04 Å (Fig. 3d). A similar confinement effect is also observed in the singlet state. The RMSD values of the isolated guests are in the range of 0.72–1.84 Å, whereas those of the doped materials decrease significantly to 0.06–0.16 Å (Fig. S37). These results demonstrate that the BP effectively suppresses conformational relaxation of the guests in both singlet and triplet excited states, thereby maintaining stable local host–guest packing during excited-state relaxation and maintaining the tightest host–guest distance for BP/MBQ. Collectively, these results highlight that spatial isomerism enables precise regulation of short-range host–guest distances, with MBQ achieving the most compact embedding in the BP lattice. These quantitative analyses substantiate that host–guest distance is the key structural determinant for TTET efficiency, confirming the rationality of the spatial isomer-directed design strategy.

Stimulus-responsive RTP materials often suffer from quenching under external stimuli, and host–guest systems typically exhibit compromised sensitivity. In this study, the electron-withdrawing pyrazine moiety in the guest molecules was employed as a chemical response site, enabling protonation/deprotonation-induced modulation of electronic properties and phosphorescent color. The TFA/TEA (selected for their high volatility) vapor-treatment procedure for all compounds solids were illustrated in Fig. S38. Upon acid–base treatment, BP emission remained unchanged, while guest molecules exhibited significant wavelength shifts, confirming that acid–base responsiveness originates from the guest (Fig. S39). Under trifluoroacetic acid (TFA) vapor, the doped system displayed a red-shifted phosphorescence with decreased intensity, which was partially reversible upon treatment with triethylamine (TEA). The incomplete reversibility likely arises from minor spatial perturbations at the host–guest interface induced by acid–base molecules. Residual acid or incomplete deprotonation may hinder the recovery of the original host–guest distance after TEA treatment. As a result, TTET is weakened to different extents, and the contribution of host phosphorescence becomes progressively more evident following acid–base treatment (Fig. 4a, b, S40 and S41). Specifically, host phosphorescence was scarcely observed in BP/MBQ, became noticeable in BP/PBQ, and finally dominated in BP/OBQ. This evolution is consistent with the decrease in TTET efficiency from MBQ to PBQ and OBQ as the host–guest distance increased. The incomplete recovery may limit applications requiring quantitative sensing or repeated switching over many cycles. Nevertheless, the pronounced and tuneable acid–base response remains suitable for qualitative visual indication, a limited number of switching cycles, and single-use information encoding.

**Fig. 4:**
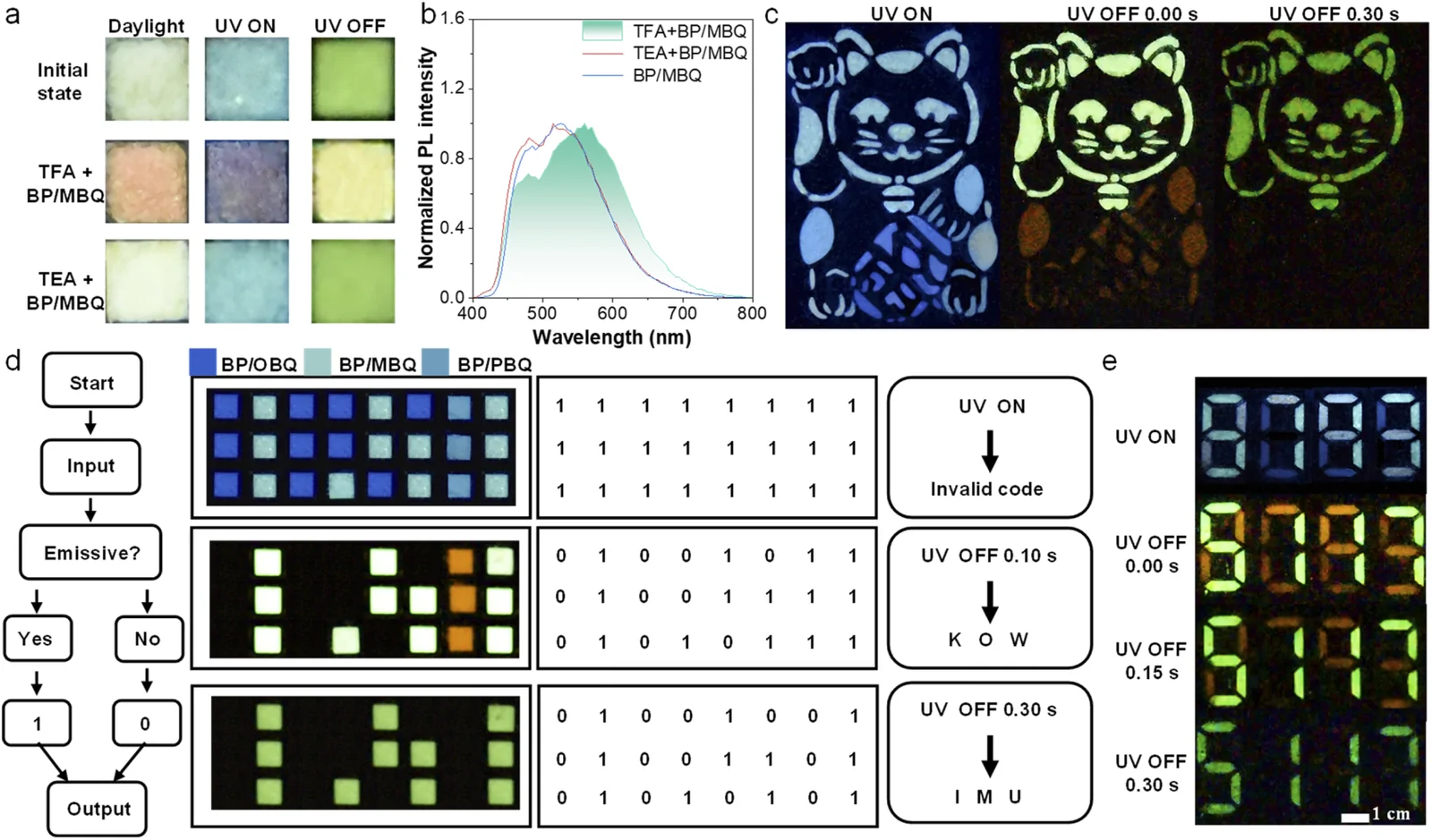
(a) Photographs of the BP/MBQ in its initial state, after protonation by TFA, and after exposure to TEA vapor, captured under daylight, UV ON and UV OFF conditions. (b) Normalized delayed emission spectra of BP/MBQ in the initial state and after TFA/TEA treatment (delay time 1 ms). (c) Photographs showing the composition of the BP/MBQ/PBQ/OBQ doped materials in the form of the “Lucky Cat” pattern. (d) Illustration of the encryption and decryption process within the time-resolved anti-counterfeiting system. (e) Photographs showing the time-resolved evolution of the “8089” digital encryption pattern.

To further explore the practical applications of these materials, their photostability and thermal stability were first evaluated. The phosphorescence intensities and lifetimes of BP/OBQ, BP/MBQ, and BP/PBQ showed no obvious decrease during continuous irradiation or after thermal treatment, demonstrating their good stability (Fig. S42 and S43). Subsequently, leveraging the distinct phosphorescence lifetimes and emission characteristics of BP/OBQ, BP/MBQ, and BP/PBQ, a “Lucky Cat” anti-counterfeiting pattern was designed. Under 365 nm UV irradiation, the pattern displays azure and dark blue fluorescence. Upon cessation of UV excitation, the cat's head exhibits long-lived green phosphorescence (BP/MBQ), whereas the body (BP/OBQ and BP/PBQ) shows short-lived orange-red emission that disappears within 0.30 s, creating a three-tiered hierarchy: “color change-short-lived quenching–long-lived persistence” (Fig. 4c). A multi-layer encryption strategy was further developed using standard 8-bit ASCII encoding, leveraging emission states (“1” for emission, “0” for non-emission) and lifetime differences (Fig. 4d). For example, at 0.10 s after removal of the UV lamp, misleading codes (“0 100 1011”, “0 100 1111”, “0 101 0111”) are generated, while at 0.30 s, the valid code “IMU” is revealed. Additionally, a “8089” digital encryption system demonstrates dynamic encryption: UV exposure shows “8089”, 0.15 s after UV off, the code transforms to “8793”, and at 0.30 s to “5117” (Fig. 4e). Compared with conventional static fluorescent patterns, the present system introduces lifetime and readout time as additional encryption dimensions. The correct information can only be decoded within a predetermined temporal window, while other time points generate misleading outputs. This temporal decoding mode adds an additional level of information protection and provides a feasible strategy for dynamic anti-counterfeiting and information encryption.

## Conclusions

This study proposes a strategy to precisely modulate host–guest intermolecular distances by leveraging the spatial isomerism of guest molecules, thereby enhancing TTET efficiency in organic RTP systems. Three pyrazine-based acceptor-triphenylamine donor isomers (OBQ, MBQ, PBQ) were doped into the BP host. Due to differences in substitution positions, these isomers exhibit distinct steric hindrance and dipole moments, leading to varied molecular embedding patterns. Such structural diversity results in significant variations in the host–guest distance. Steady-state and delayed emission spectra reveal that with increasing guest doping ratio, the intrinsic phosphorescence of BP is progressively quenched, while guest phosphorescence is concurrently enhanced, confirming efficient TTET within the doped systems. Notably, molecular analysis shows that MBQ exhibits optimal host–guest compatibility with multiple parameters—including molecular distortion, dipole moment (2.31 D), and surface electrostatic potential difference (ΔESP = 56.88 kcal mol^−1^)—highly matching those of BP (3.13 D, 56.51 kcal mol^−1^). PXRD confirmed that none of the dopants disrupt the host crystal framework, indicating that the observed differences in RTP performance primarily arise from variations in local intermolecular geometry rather than from overall lattice distortion. QM/MM simulations directly reveal that MBQ exhibits the strongest intermolecular interactions and the shortest host–guest average distance (2.86 Å), compared to 3.26 Å for BP/PBQ and 3.36 Å for BP/OBQ. This structural feature correlates directly with its highest phosphorescence quantum yield and lifetime (*Φ*_Phos._ = 8.97%, *τ*_P_ = 158 ms). In addition, all doped systems display distinct acid/base-responsive behavior, enabling reversible control of phosphorescent color. This endows the system with distance sensitivity and stimulus-responsive phosphorescence, enabling tri-mode regulation of color, brightness, and lifetime. Collectively, this work not only systematically elucidates the regulatory mechanism of RTP based on the intermolecular distance control but also provides an efficient and tunable design strategy for RTP materials, thereby expanding the potential applications of organic phosphorescent materials in information security and intelligent responsive systems.

## Author contributions

J. W., G. J. and B. T. initiated and supervised the project. H. S. conceived the research idea and developed the methodology. H. S., C. L., J. G., N. W., R. P., J. T. and X. X. designed and performed the simulations and experiments. H. S. constructed the experimental system. H. S., C. L., G. J. and J. W. analysed the data and interpreted the results. H. S. wrote the original draft. H. S., G. J. and J. W. revised the manuscript with input from all authors. All authors discussed the results and approved the final version of the manuscript.

## Conflicts of interest

There are no conflicts to declare.

## Supplementary Material

SC-017-D6SC04960E-s001

SC-017-D6SC04960E-s002

SC-017-D6SC04960E-s003

SC-017-D6SC04960E-s004

SC-017-D6SC04960E-s005

## Data Availability

CCDC 2545022, 2545024 and 2545026 contain the supplementary crystallographic data for this paper.^61*a*–*c*^ The data supporting this article, including experimental procedures, synthetic details, characterization data, photophysical measurements, crystallographic analysis, and computational methods, are provided in the main article and the supplementary information (SI). Supplementary information is available. See DOI: https://doi.org/10.1039/d6sc04960e.
