# Supplementary material for: Spatial isomerism regulates host–guest distance for highly efficient organic room-temperature phosphorescence
Source: Chem Sci. 2026 Aug 3;17(38):18238–47. doi: 10.1039/d6sc04960e (PMC13458974; doi:10.1039/d6sc04960e)
Supplement: Supplementary file 1 [file SC-017-D6SC04960E-s004.pdf]

Supplementary Information (SI) for Chemical Science.
This journal is © The Royal Society of Chemistry 2026

Supporting Information

Spatial Isomerism Regulates Host–Guest Distance for Highly Efficient Organic Room-Temperature Phosphorescence

Hao Sun,[†a] Chen Lu,[† a] Jianye Gong,[a] Nuan Wen,[a] Ru Pang,[a] Jin Tao,[a] Xiaolang Xiong,[a] Guoyu Jiang,*[a] Ben Zhong Tang,*[b] and Jianguo Wang*[a]

[a]College of Chemistry and Chemical Engineering, Institute of Green Chemistry and Environment, Institutes of Biomedical Sciences, Inner Mongolia Key Laboratory of Synthesis and Application of Organic Functional Molecules, Inner Mongolia University, Hohhot 010021, P. R. China.

E-mail: jiangguoyu@mail.ipc.ac.cn (G. Jiang); wangjg@iccas.ac.cn (J. Wang)

[b]Guangdong Basic Research Center of Excellence for Aggregate Science, School of Science and Engineering, The Chinese University of Hong Kong (Shenzhen), Longgang, Shenzhen, Guangdong 518172, P. R. China.

E-mail: tangbenz@cuhk.edu.cn (B. Z. Tang)

[†]Equally contributed to this work.

Table of Contents

Experimental Procedures S4

Materials S4

Instruments S4

Density Functional Theory Calculations S4

Crystal Growth S5

Preparation of Doped Materials S5

Synthesis and Characterization S6

Figure S1 .... S6

Figure S2 .... S6

Figure S3 .... S7

Figure S4 .... S8

Figure S5 .... S8

Figure S6 .... S9

Figure S7 .... S9

Figure S8 .... S10

Figure S9 .... S10

Figure S10 .... S11

Figure S11 .... S11

Figure S12 .... S12

Figure S13 .... S12

Figure S14 .... S13

Table S1 .... S13

Figure S15 .... S14

Figure S16 .... S14

Figure S17 .... S14

Figure S18 .... S15

Figure S19 .... S15

Figure S20 .... S15

Figure S21 .... S16

Figure S22 .... S16

Figure S23 .... S16

Figure S24 .... S17

Figure S25 .... S17

Figure S26 .... S17

Figure S27 .... S18

Figure S28 .... S18

Figure S29 .... S19

Figure S30 .... S19

Table S2 .... S20

Figure S31 .... S20

Figure S32 .... S21

Figure S33 .... S21

Figure S34 .... S21

Figure S35 .... S22

Figure S36 .... S22

Figure S37 .... S22

Figure S38 .... S23

Figure S39 .... S23

Figure S40 .... S23

Figure S41 .... S24

Figure S42 .... S24

Figure S43 .... S24

Notes and References S25

Experimental Procedures

Materials.

Benzophenone of sublimation grade was purchased from Aladdin. N,N-Diphenyl-4-(4,4,5,5-tetramethyl-1,3,2-dioxaborolan-2-yl)aniline was purchased from Soochiral chemistry. 2,5-Dibromopyrazine, 2,6-dibromopyrazine, and 2,3-dibromopyrazine were purchased from Adamas. All other chemicals and reagents were purchased from Energy-Chemical, Sigma Aldrich, J&K without additional treatment before use.

Instruments.

The structure and purity of the synthesized organic compounds were characterized by ^{1}H and ^{13}C NMR spectroscopy using Bruker ARX 600 spectrometers. Chloroform-*d* and dimethyl sulfoxide-d_6 were used as solvents, with tetramethylsilane (TMS) as the internal reference. High-resolution mass spectra (HRMS) were measured with a LCMS9030 spectrometer. Absorption spectra were measured on a SHIMADZU UV-2600i spectrophotometer. Photoluminescence (PL) spectra were recorded on a FS5 Spectrofluorometer. Single crystal X-ray diffraction was performed on a Rigaku Oxford Diffraction Supernova Dual Source, Cu at Zero equipped with an AtlasS2 CCD using Cu Kα radiation. The powder X-ray diffraction data were conducted on a SmartLab SE using Cu-Kα radiation from 5° to 40°. The absolute fluorescence quantum yield was measured using a Hamamatsu quantum yield spectrometer C11347 Quantaurus QY. High performance liquid chromatography (HPLC) was performed on a chromAssist chromatography workstation using a HITACHI LaChrom C18. The photos were taken by Nikon D750.

Density Functional Theory Calculations.

The quantum chemical calculations were performed with density functional theory (DFT) and time-dependent density functional theory (TD-DFT) implemented the programs Gaussian 09 [1] and ORCA 5.0. [2] Based on the optimized geometries, TD-DFT was utilized at the same level to calculate the excitation energies in the singlet (S_n) and triplet (T_n) states of monomer. The natural transition orbitals (NTO) analysis was further carried out by Multiwfn [3] to obtain the main NTO contributing to the electron transition. The electrostatic potential (ESP) characteristic parameters were calculated based on the single crystal structure with electronic wave function information using Multiwfn. The electron density analysis was implemented on Multiwfn. The corresponding structure and isosurfaces were generated with the program VMD [4].

All atomic and molecular dynamics simulations were performed using the GAFF force field in the GROMACS-2019.6 package, with molecular charges for BP and OBQ obtained via RESP fitting. Van der Waals interactions and long-range electrostatic interactions were calculated using cutoff distances of 1.2 nm, with the latter treated by the particle-mesh Ewald (PME) method. To simulate the aggregation state, 200 molecules were randomly placed in a 4×4×6 nm^3 simulation box. The system was compressed for 5 ns at 600 K and 100 bar, followed by a 10 ns simulation at 600 K and 1 bar. A gradual cooling from 600 K to 298 K over 3 ns was then performed, ending with a 20 ns equilibration simulation at 298 K and 1 bar. Temperature and pressure were controlled using the V-rescale thermostat and Berendsen barostat, while the final equilibration employed the V-rescale

thermostat and Parrinello-Rahman barostat to achieve equilibrium configurations. The QM/MM-optimized Cartesian coordinates of the three host–guest models are provided as separate Supporting Information files.

Initial BP/guest models for BP/OBQ, BP/MBQ, and BP/PBQ were constructed from the BP crystal structure by replacing selected BP molecules with the corresponding guest molecule in each case, giving host–guest systems with a guest–host molecular ratio of 1:191. Starting from this configuration, we carried out a 5 ns production molecular dynamics (MD) simulation of the full system using the GROMACS software package to achieve structural equilibration. After completing the MD simulations, the final host–guest aggregate cluster configurations were subjected to hybrid quantum mechanics/molecular mechanics (QM/MM) calculations using a two-layer ONIOM model. The theoretical approach for the QM/MM calculations was consistent with the method used in the crystal structure calculations. The ONIOM models for host-guest doping system were partitioned into two layers. The guest molecule in the host–guest system and in the center of guest crystal was selected as high layer and treated by B3LYP functional with the 6-31G* basis set, and other molecules were set as low-layer and treated by universal force field. The QM region provides key information of the electronic excited states, while the MM region includes environmental effects.

Crystal Growth.

The single crystals of OBQ, MBQ and PBQ were cultured through slow solvent evaporation using dichloromethane (DCM)/n-hexane as the solvent.

Preparation of Doped Materials.

Place a certain amount of pre-weighed host and guest together and heat them in the ambient environment to 60 °C. After the host is completely melted, slowly stir with a glass rod until the guest is dissolved in the melted host, then the mixed system is cooled to room temperature, and the mixed system is crystallized to obtain the doped material. The doped materials with high host–guest mass ratio (200:1–1000:1) are using direct weighing method. While, for the doped materials with a low mass ratio of host–guest (2000:1–100000:1), indirect dilution method is required, such as continuing to mix the doped material (1:100) with the corresponding amount of pure host.

Synthesis and Characterization

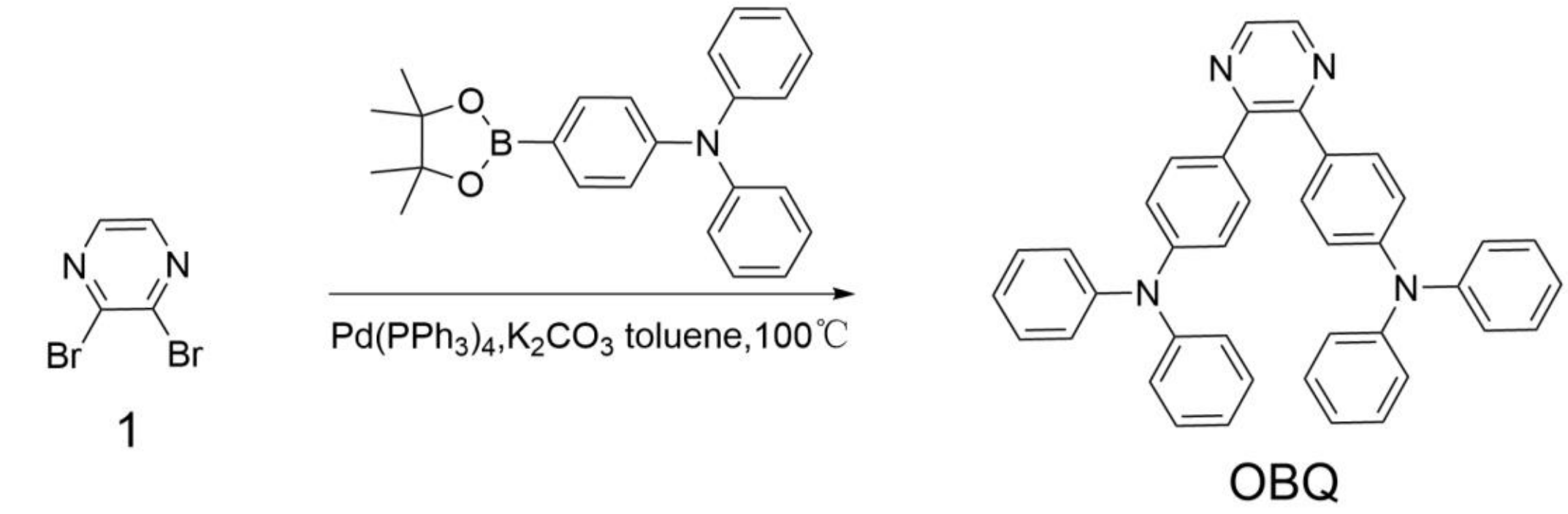

Figure S1. Synthetic procedure for compound OBQ.

Synthesis of OBQ: To a 100.0 mL two-neck flask equipped with a condenser was added 2,3-dibromopyrazine (compound **1**, 500.0 mg, 2.1 mmol), N,N-diphenyl-4-(4,4,5,5-tetramethyl-1,3,2-dioxaborolan-2-yl)aniline (2000.0 mg, 5.4 mmol) and $Pd(PPh_3)_4$ (438.0 mg, 0.3 mmol). The solids were placed under an atmosphere of N_2. Freshly distilled toluene (20.0 mL) and 4.0 M aq. K_2CO_3 (2.2 mL) was added via syringe. The reaction mixture was refluxed for 16 h at 100 °C. Then, the reaction was cooled to room temperature. The resulting slurry was suspended in CH_2Cl_2 (115.0 mL) and washed with H_2O (3 × 75.0 mL) followed by saturated aq. NH_4Cl (3 × 35.0 mL). The organic layer was collected, dried over Na_2SO_4, filtered, and concentrated under reduced pressure. The crude product was purified by silica gel column chromatography using petroleum ether/ethyl acetate (*v*:*v*, 30:1) as an eluent to afford a cyan solid (442.4 mg, 37% yield). ^1H NMR (600 MHz, DMSO-*d*6) *δ* 8.59 (s, 2H), 7.34 – 7.28 (m, 12H), 7.09 (d, *J* = 6.6 Hz, 4H), 7.04 (d, *J* = 6.8 Hz, 8H), 6.89 (d, *J* = 8.1 Hz, 4H). ^{13}C NMR (151 MHz, Chloroform-*d*) *δ* 152.20, 148.45, 147.31, 141.24, 131.84, 130.47, 129.34, 124.98, 123.45, 122.16. HRMS: m/z: $[M+H]^+$ calcd: 567.2549, found: 567.2544.

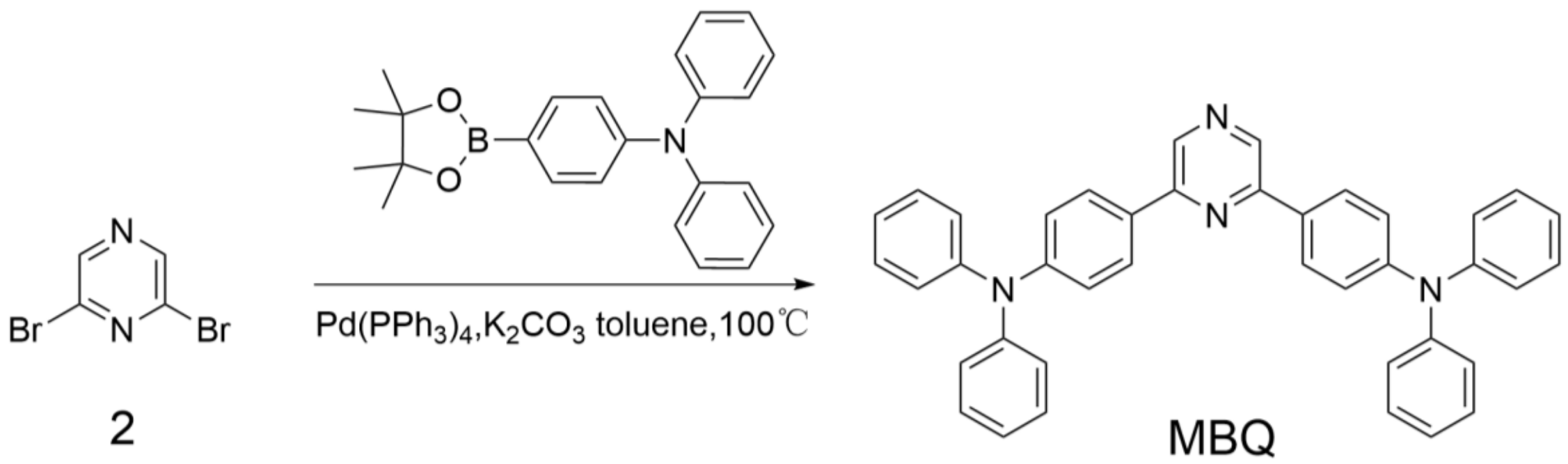

Figure S2. Synthetic procedure for compound MBQ.

Synthesis of MBQ: To a 100.0 mL two-neck flask equipped with a condenser was added 2,6-dibromopyrazine (compound **2**, 500.0 mg, 2.1 mmol), N,N-diphenyl-4-(4,4,5,5-tetramethyl-1,3,2-dioxaborolan-2-yl)aniline (2000.0 mg, 5.4 mmol) and $Pd(PPh_3)_4$ (438.0 mg, 0.3 mmol). The solids were placed under an atmosphere of N_2. Freshly distilled toluene (20.0 mL) and 4.0 M aq. K_2CO_3 (2.2 mL) was added via syringe. The reaction mixture was refluxed for 16 h at 100 °C. Then, the reaction was cooled to room temperature. The resulting slurry was suspended in CH_2Cl_2 (115.0 mL) and washed with H_2O (3 × 75.0 mL) followed by saturated aq. NH_4Cl (3 × 35.0 mL). The organic layer was collected, dried over Na_2SO_4, filtered, and concentrated under reduced pressure.

The crude product was purified by silica gel column chromatography using petroleum ether/ethyl acetate (*v*:*v*, 50:1) as an eluent to afford a light-yellow solid (669.6 mg, 56% yield). ^{1}H NMR (600 MHz, Chloroform-*d*) *δ* 8.80 (s, 2H), 7.99 (d, *J* = 8.6 Hz, 4H), 7.30 (t, *J* = 7.8 Hz, 8H), 7.16 (d, *J* = 7.9 Hz, 12H), 7.09 (t, *J* = 7.3 Hz, 4H). ^{13}C NMR (151 MHz, Chloroform-*d*) *δ* 151.28, 149.60, 147.24, 138.01, 129.75, 129.42, 127.84, 125.10, 123.67, 122.64. HRMS: m/z: $[M+H]^{+}$ calcd: 567.2549, found: 567.2542.

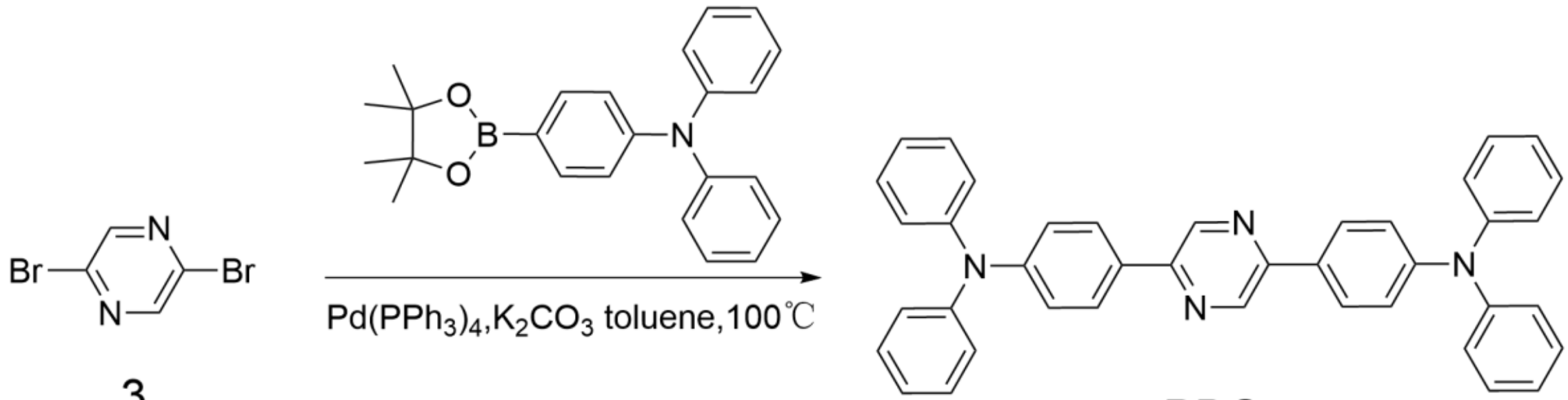

3

PBQ

Figure S3. Synthetic procedure for compound PBQ.

Synthesis of PBQ: To a 100.0 mL two-neck flask equipped with a condenser was added 2,5-dibromopyrazine (compound **3**, 500.0 mg, 2.1 mmol), N,N-diphenyl-4-(4,4,5,5-tetramethyl-1,3,2-dioxaborolan-2-yl)aniline (2000.0 mg, 5.4 mmol) and $Pd(PPh_3)_4$ (438.0 mg, 0.3 mmol). The solids were placed under an atmosphere of N_2. Freshly distilled toluene (20.0 mL) and 4.0 M aq. K_2CO_3 (2.2 mL) was added via syringe. The reaction mixture was refluxed for 16 h at 100 °C. Then, the reaction was cooled to room temperature. The resulting slurry was suspended in CH_2Cl_2 (115.0 mL) and washed with H_2O (3 × 75.0 mL) followed by saturated aq. NH_4Cl (3 × 35.0 mL). The organic layer was collected, dried over Na_2SO_4, filtered, and concentrated under reduced pressure. The crude product was purified by silica gel column chromatography using petroleum ether/ethyl acetate (*v*:*v*, 20:1) as an eluent to afford a yellowish green solid (717.4 mg, 60% yield). ^{1}H NMR (600 MHz, Chloroform-*d*) *δ* 8.97 (s, 2H), 7.93 (d, *J* = 8.3 Hz, 4H), 7.30 (t, *J* = 7.5 Hz, 8H), 7.16 (d, *J* = 7.7 Hz, 12H), 7.09 (t, *J* = 7.1 Hz, 4H). ^{13}C NMR (151 MHz, Chloroform-*d*) *δ* 149.40, 147.24, 140.41, 129.42, 127.48, 125.10, 123.66, 122.76. HRMS: m/z: $[M+H]^{+}$ calcd: 567.2549, found: 567.2542.

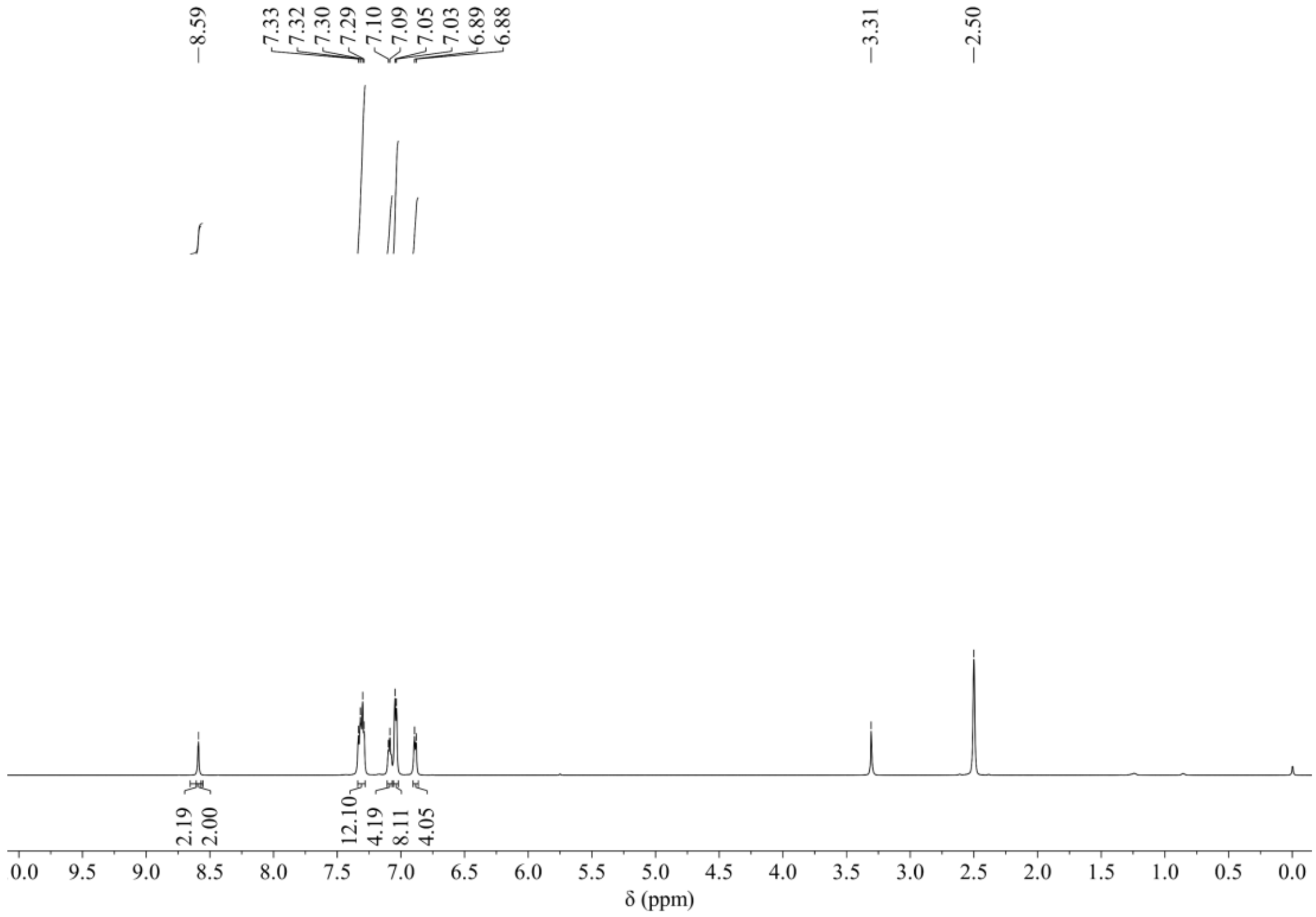

Figure S4. ^{1}H NMR spectrum of OBQ in DMSO-d_6.

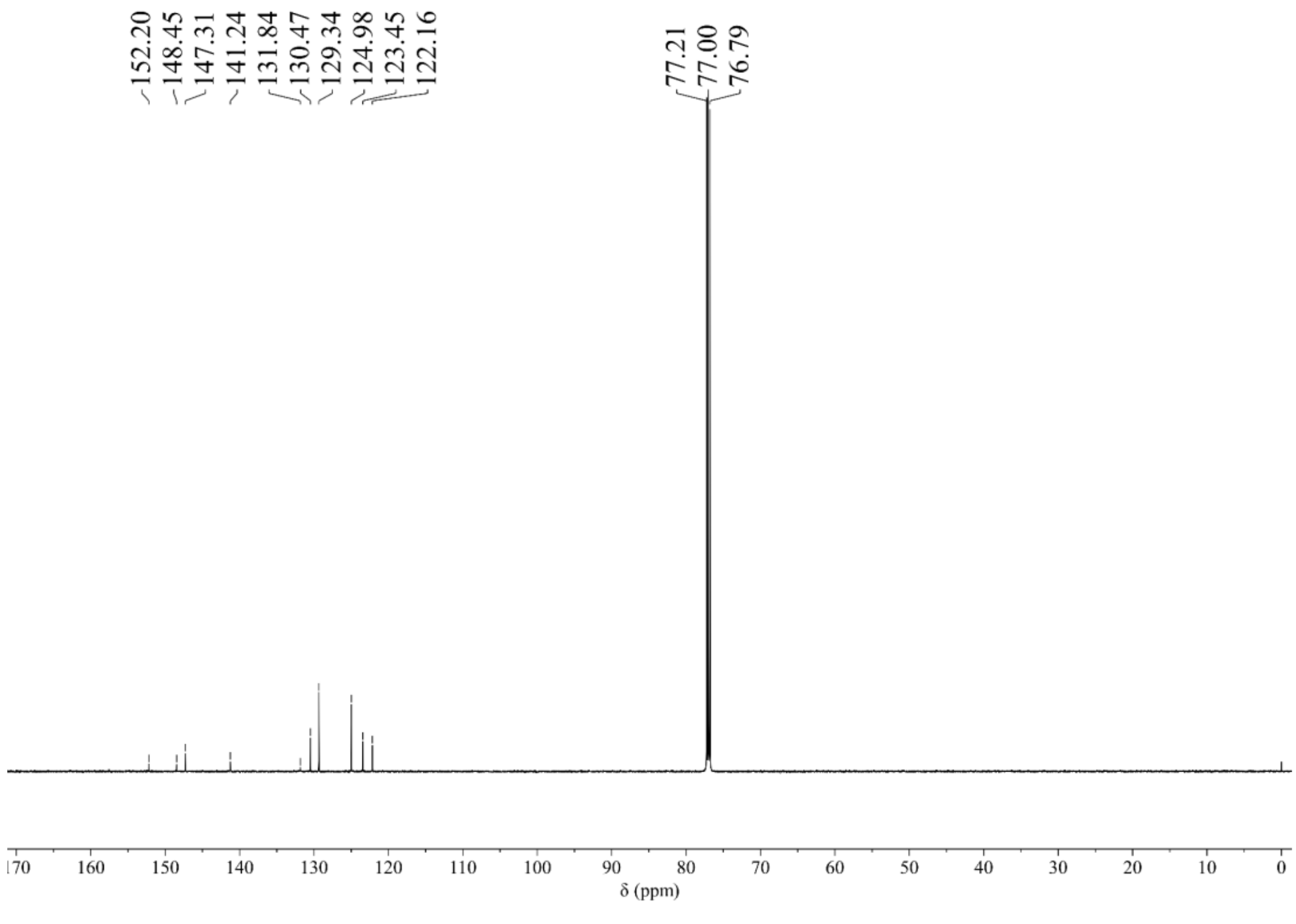

Figure S5. ^{13}C NMR spectrum of OBQ in $CDCl_3$.

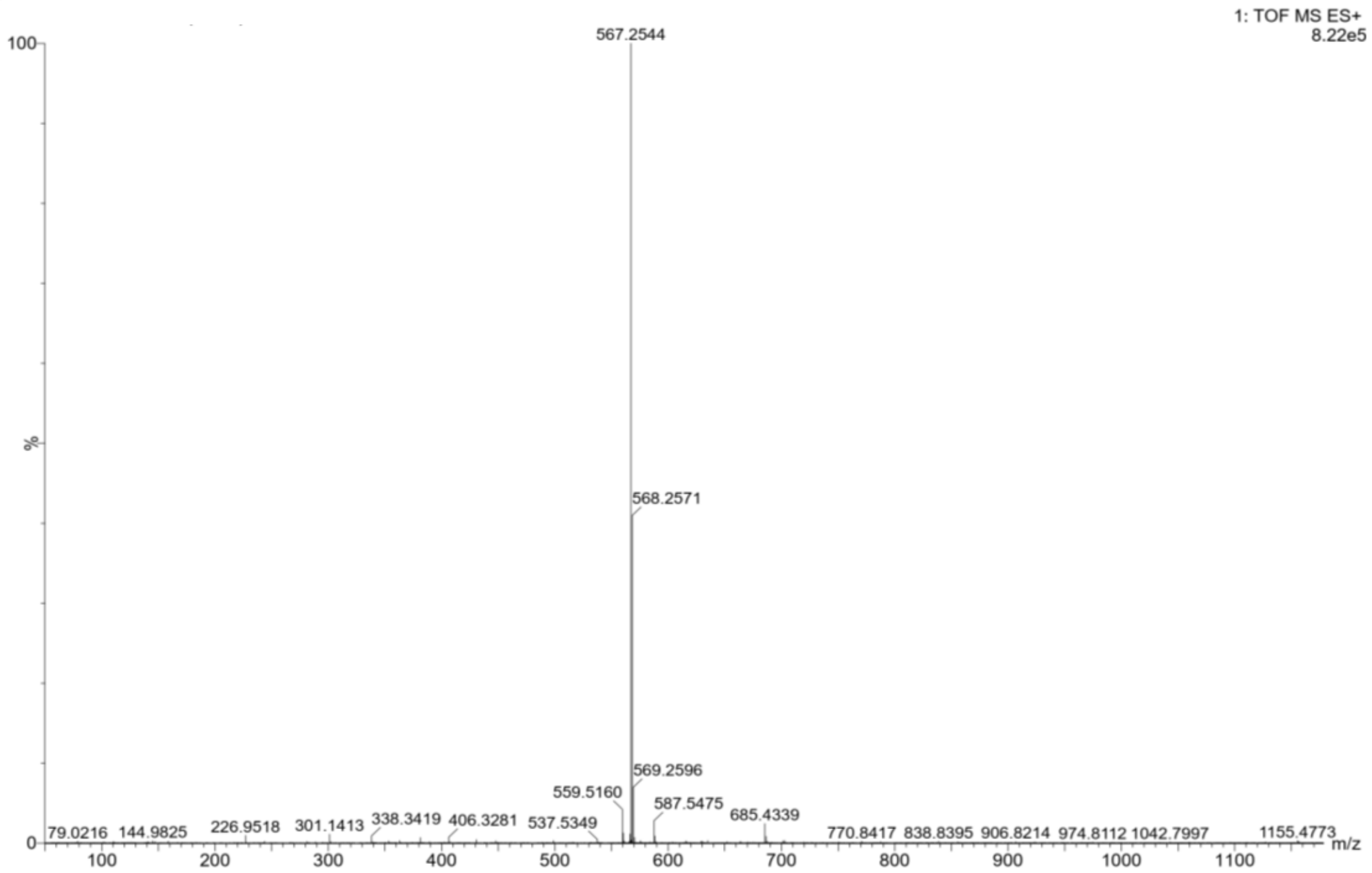

Figure S6. High-resolution mass spectrum of OBQ.

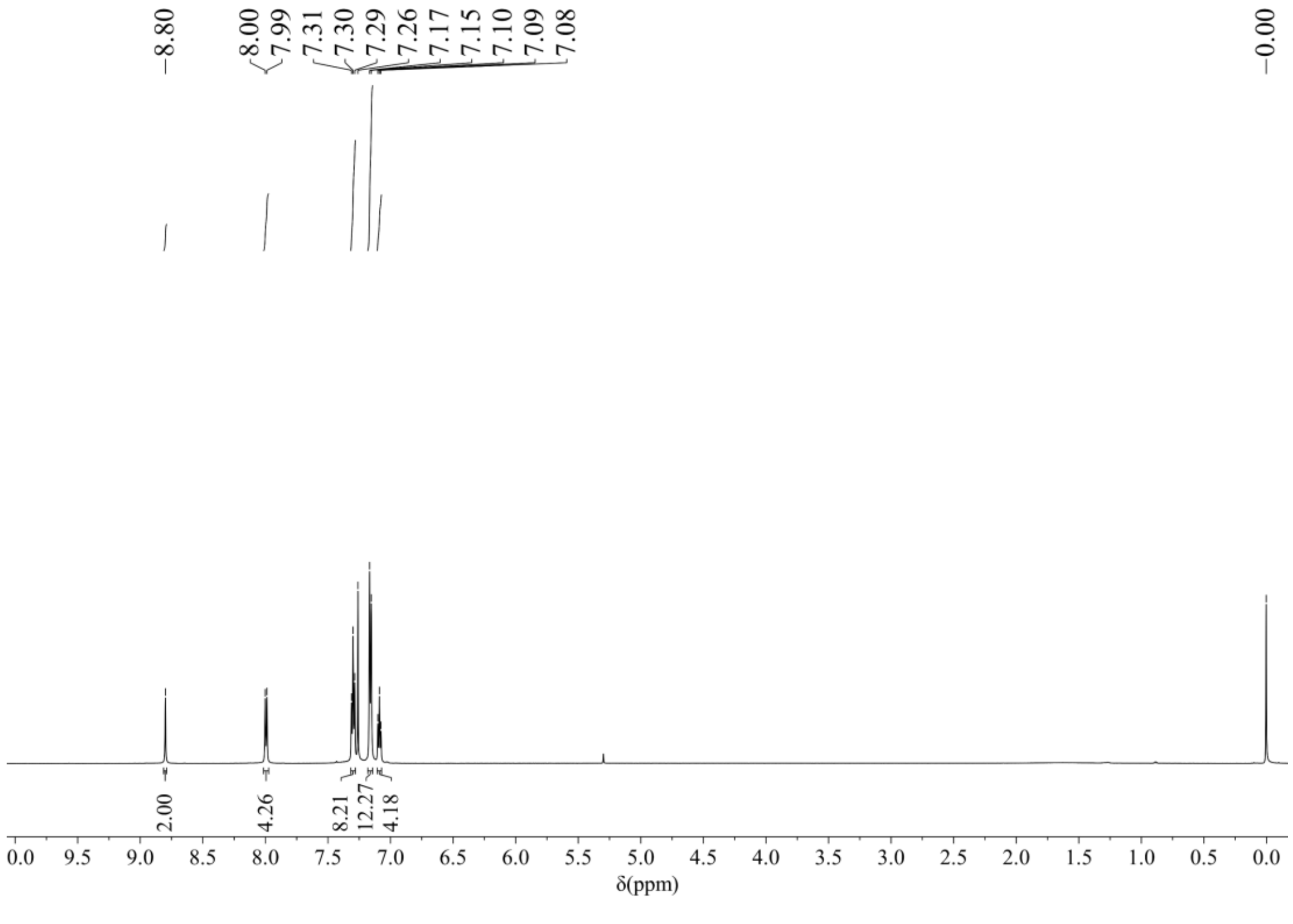

Figure S7. ^1H NMR spectrum of MBQ in $CDCl_3$.

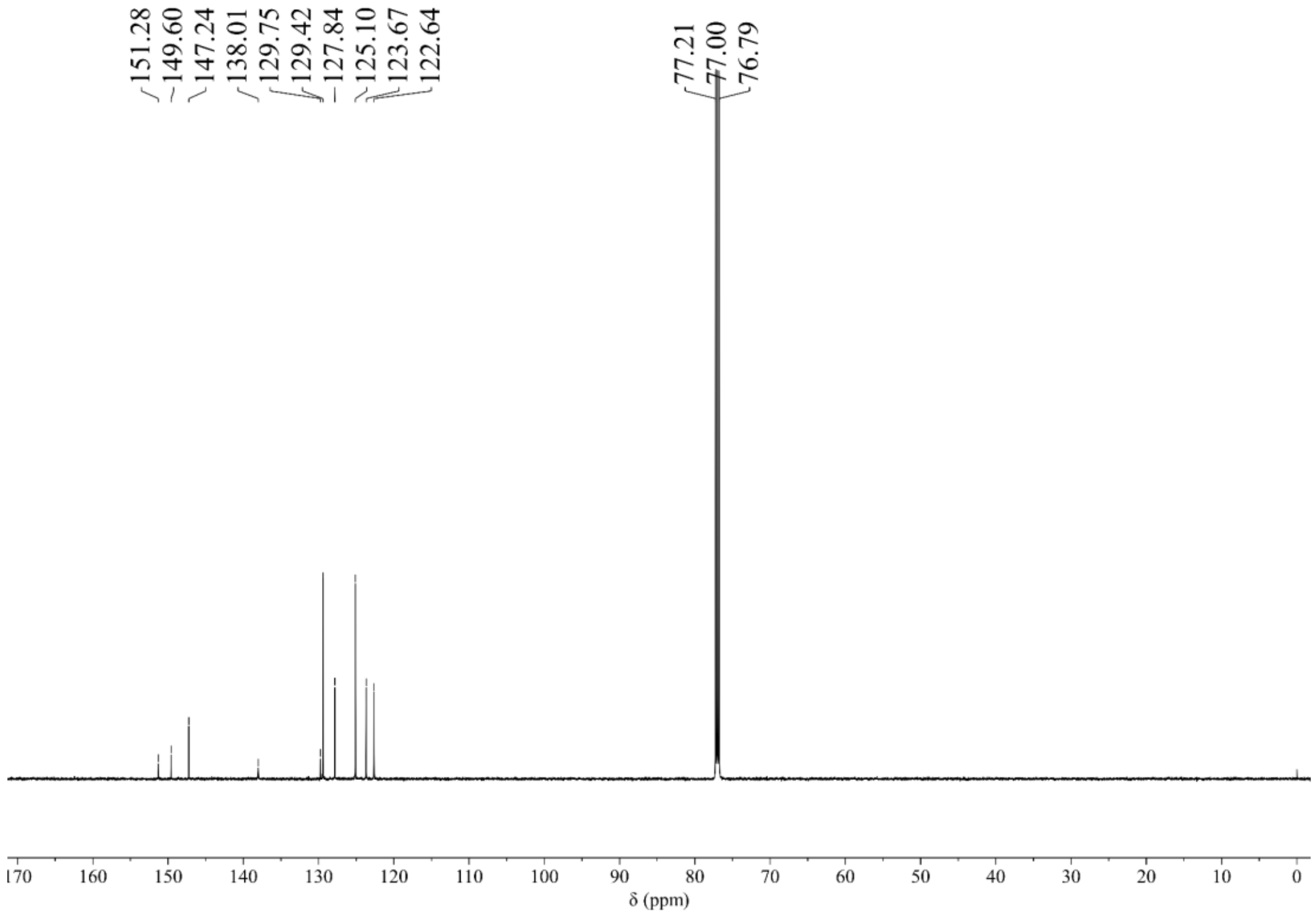

Figure S8. ^{13}C NMR spectrum of MBQ in $CDCl_3$.

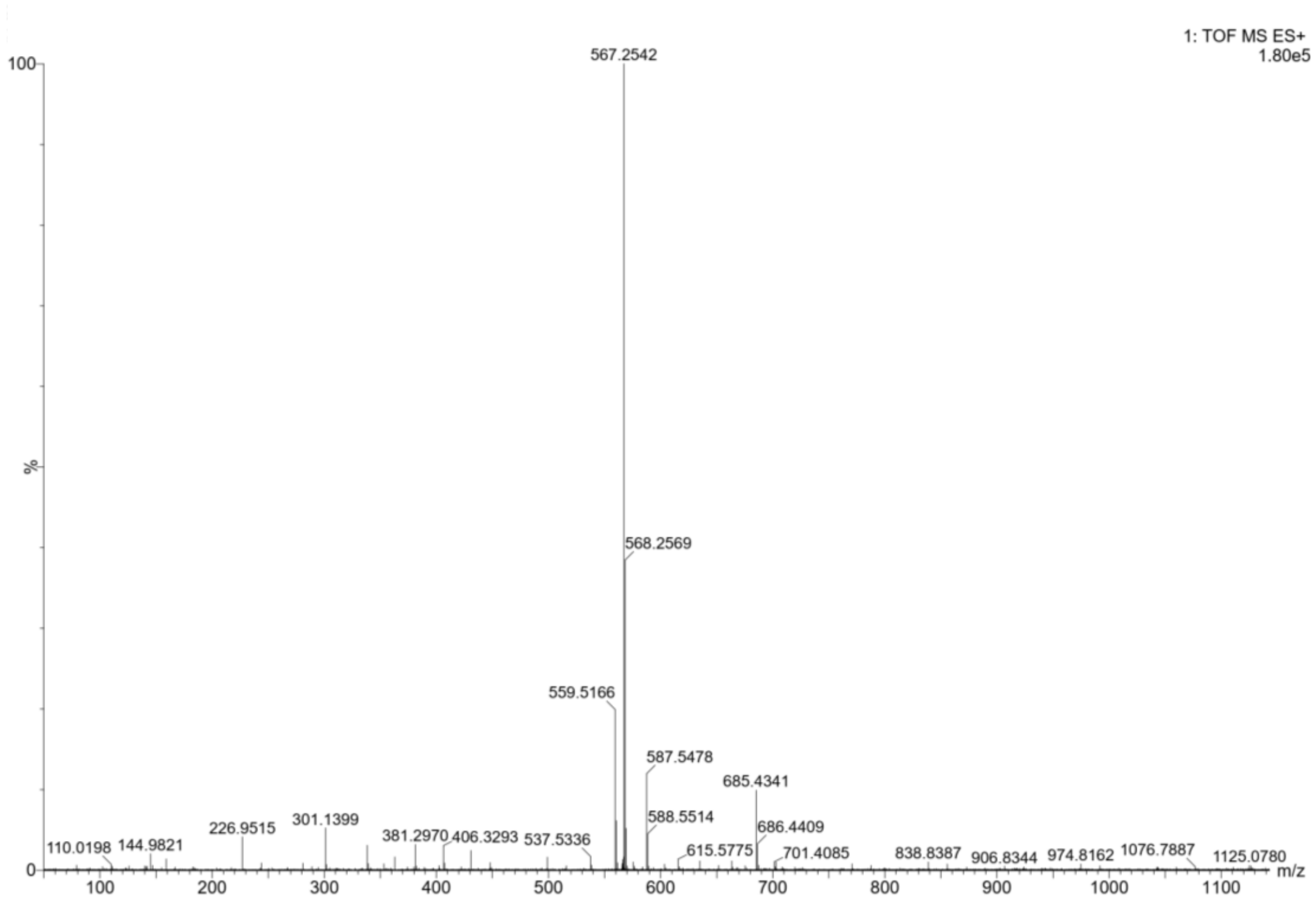

Figure S9. High-resolution mass spectrum of MBQ.

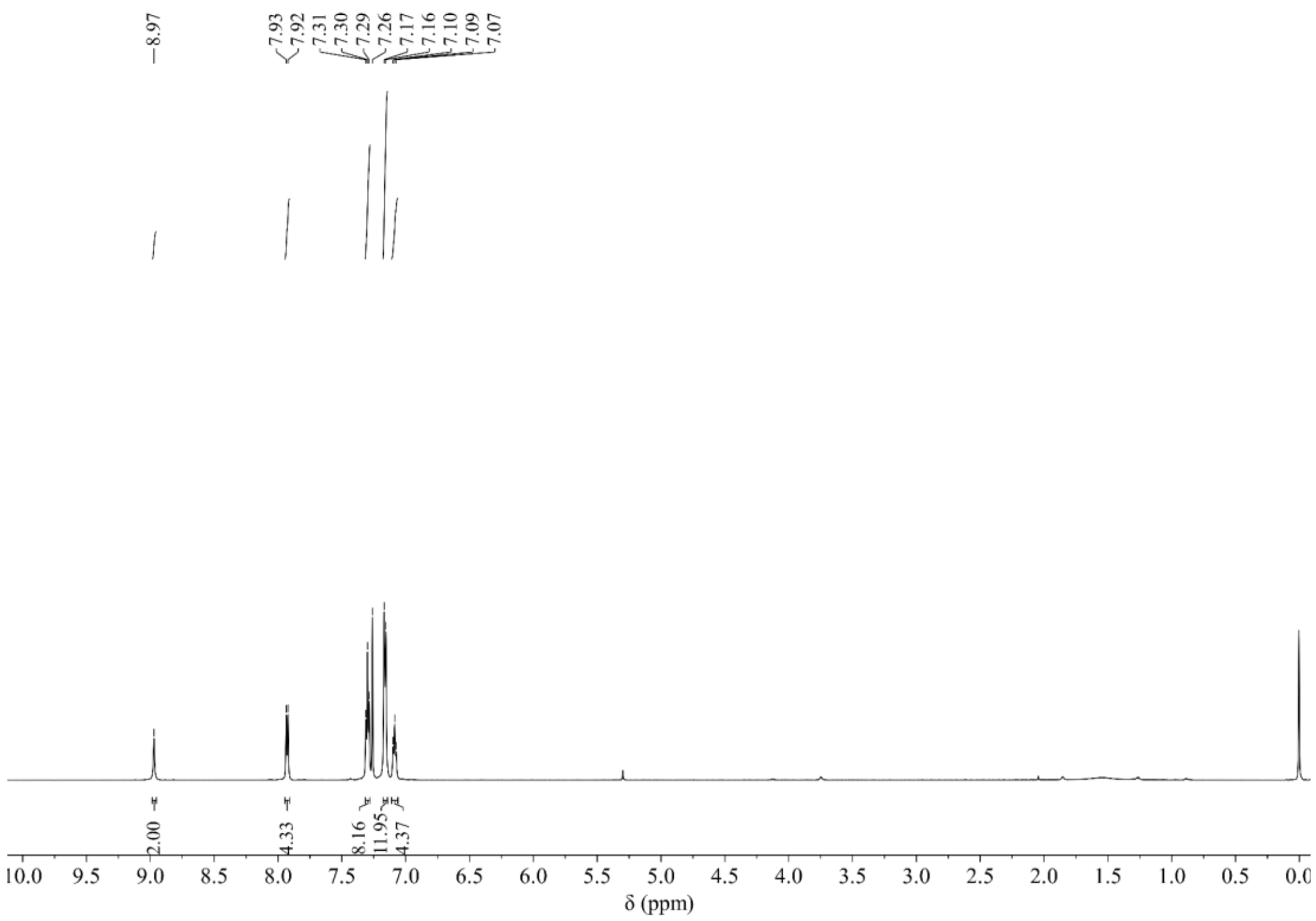

Figure S10. ^{1}H NMR spectrum of PBQ in $CDCl_3$.

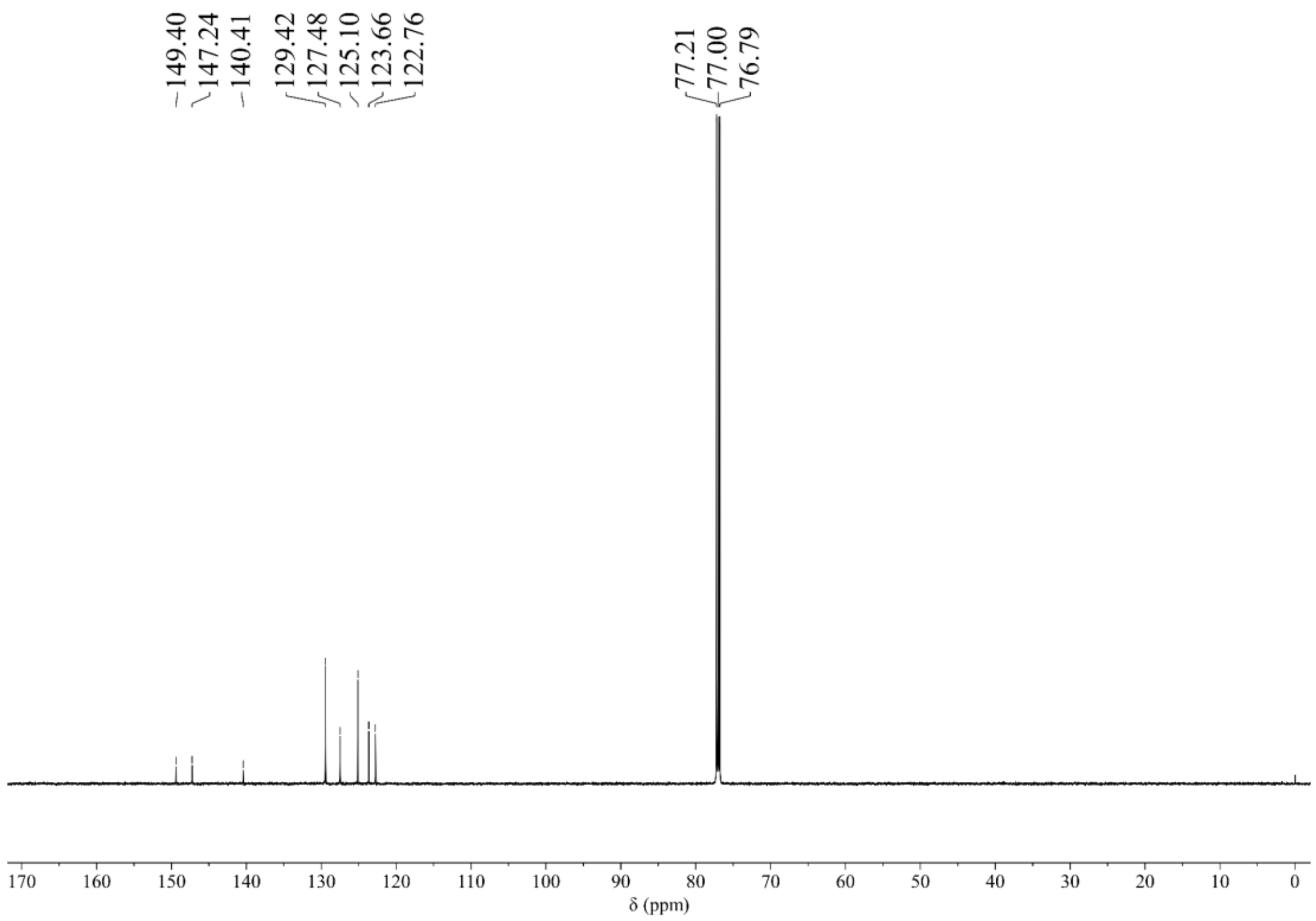

Figure S11. ^{13}C NMR spectrum of PBQ in $CDCl_3$.

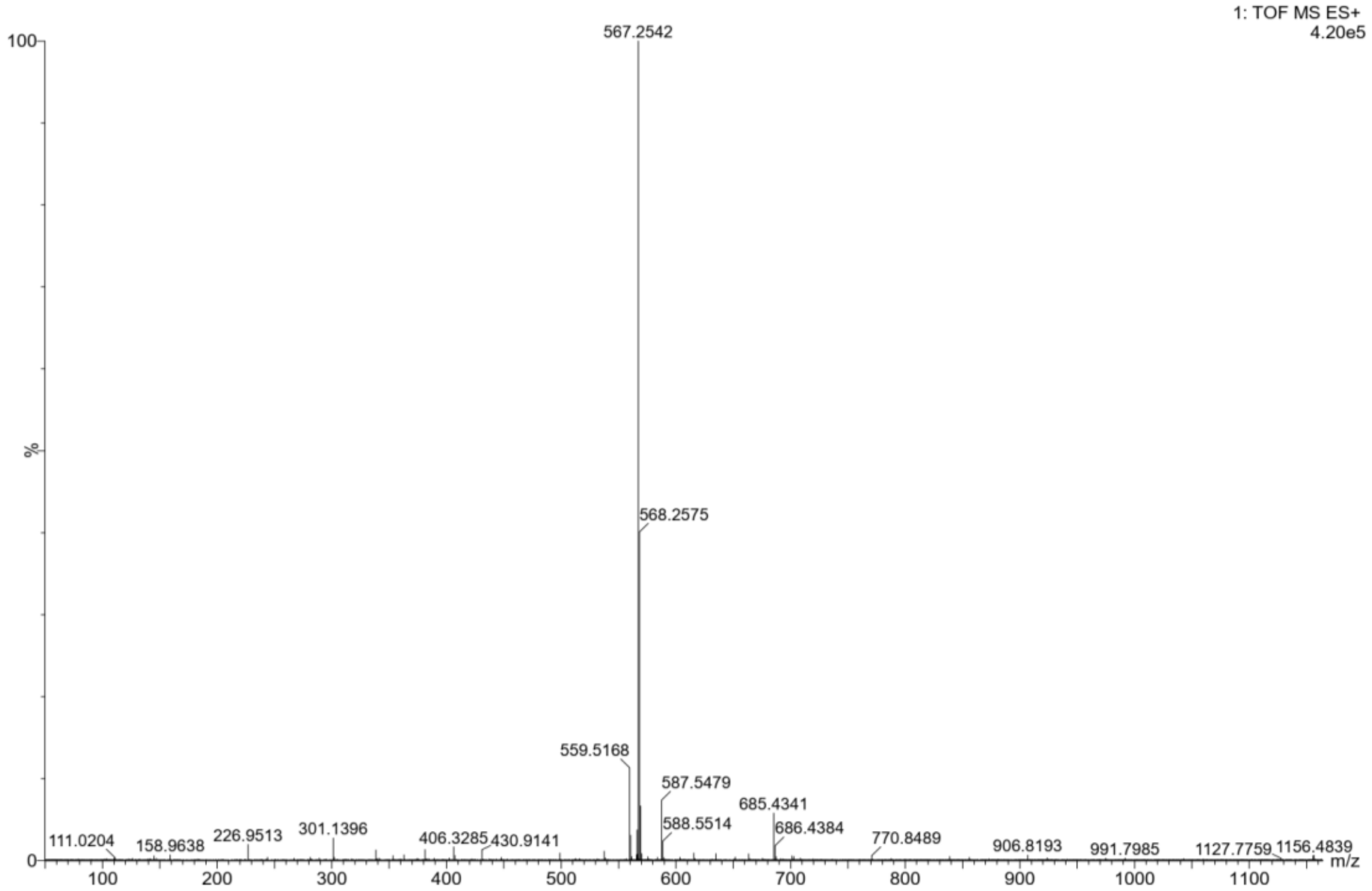

Figure S12. High-resolution mass spectrum of PBQ.

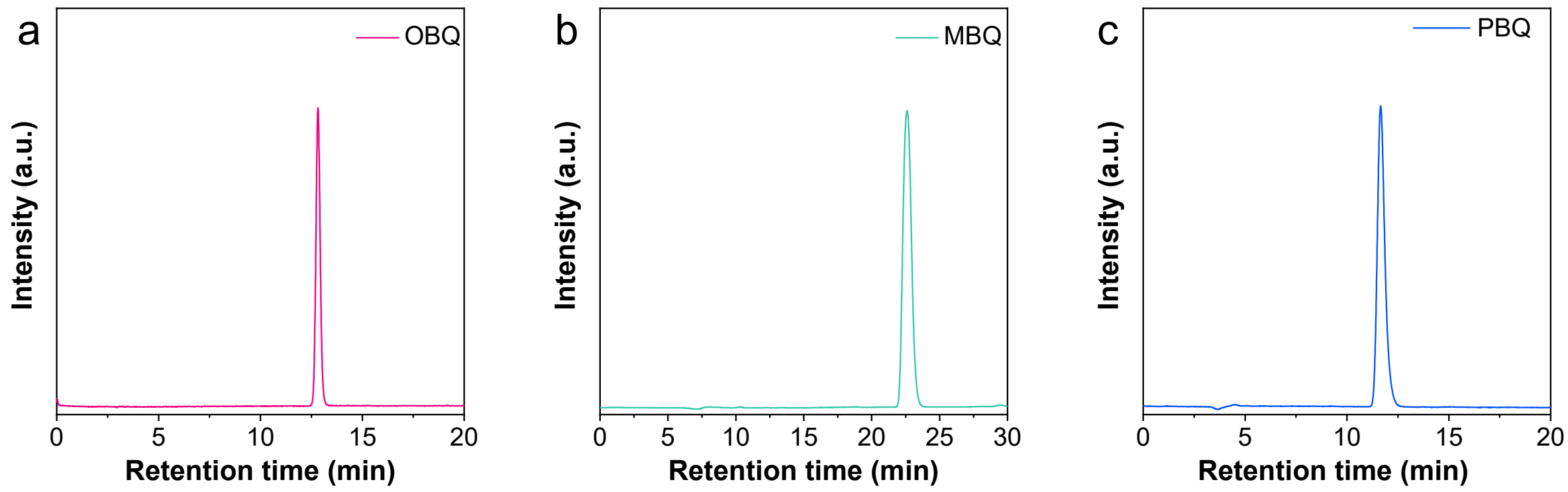

Figure S13. HPLC spectra of OBQ (a), MBQ (b), and PBQ (c). Eluent: methanol/acetonitrile = 7:3. Flow rate: 1 mL/min.

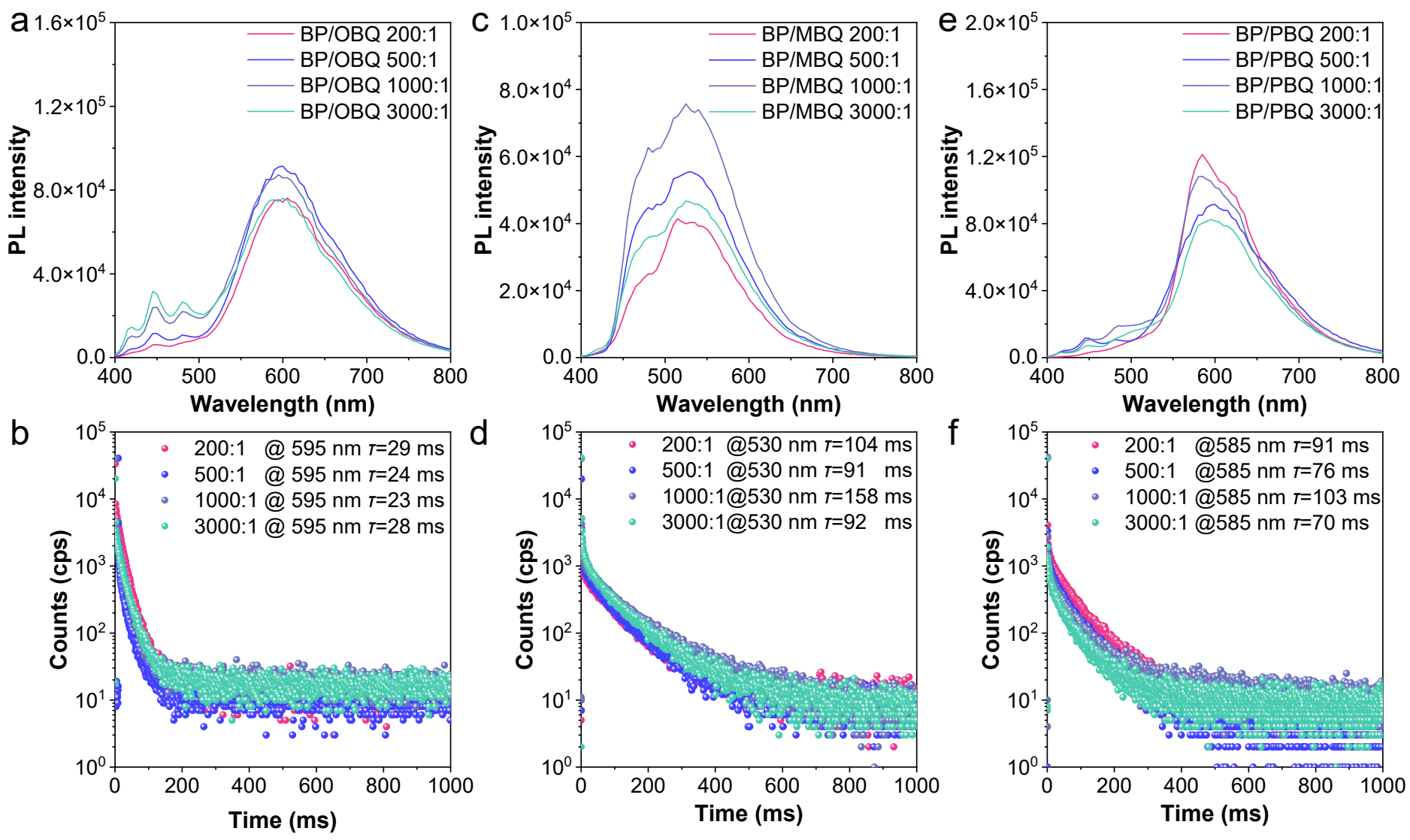

Figure S14. The delayed emission spectra (a) and time-resolved emission decay curves (b) of BP/OBQ at different mass ratios. The delayed emission spectra (c) and time-resolved emission decay curves (d) of BP/MBQ at different mass ratios. The delayed emission spectra (e) and time-resolved emission decay curves (f) of BP/PBQ at different mass ratios. Excitation wavelength: λ_{ex}: 360 nm. Delay time 1 ms.

Table S1. Photophysical data of the host–guest materials.

Doping system	Doping ratio	λ_F[a] (nm)	λ_P[b] (nm)	τ_P[c] [ms]	Φ_{total}[d] [%]	$\Phi_{Phos.}$[e] [%]
BP/OBQ	200:1	450	595	29	5.35	0.63
	500:1	450	595	24	4.45	0.64
	1000:1	447/477	595	23	3.52	0.81
	3000:1	450/477	595	28	3.42	0.43
BP/MBQ	200:1	455/530	530	104	7.43	3.97
	500:1	470/530	530	91	8.41	6.17
	1000:1	480/530	530	158	10.13	8.97
	3000:1	470/530	530	92	3.55	2.02
BP/PBQ	200:1	490/585	585	91	7.29	1.71
	500:1	480/585	585	76	5.28	1.22
	1000:1	445/475	585	103	5.68	1.44
	3000:1	445/475	585	70	4.32	0.74

[a] Steady-state emission maxima of the doped materials. [b] The phosphorescence emission of the doped materials. [c] The phosphorescence lifetime at the optimal emission wavelength. [d] Total photoluminescence quantum yields of the doped materials. [e] Phosphorescence quantum yields of doped materials. Excitation wavelength: λ_{ex}: 360 nm. Delay time 1 ms.

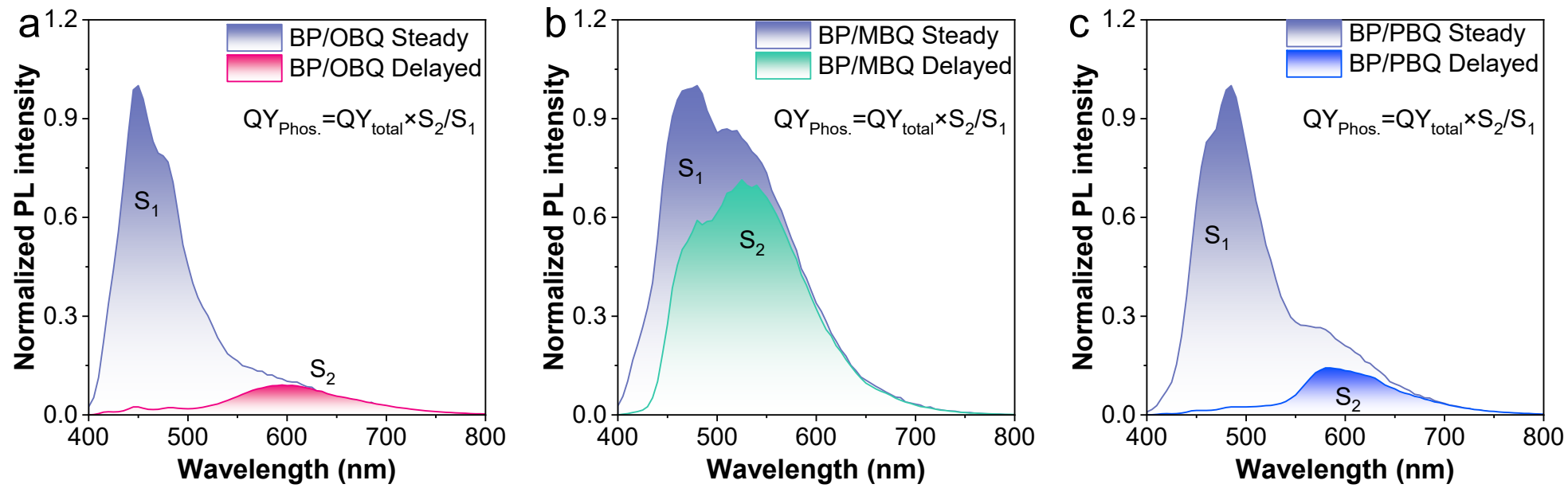

Figure S15. The phosphorescence quantum yields were calculated according to previous report[5] from the area ratios of steady-state and delayed emission spectra of BP/OBQ (a), BP/MBQ (b), BP/PBQ (c). S_1 denotes the integrated area of the total photoluminescence component, and S_2 denotes that of the phosphorescence component. Host–guest doping ratio was 1000:1. Excitation wavelength: λ_{ex}: 360 nm. Delay time 1 ms. $QY_{Phos.}$: phosphorescence quantum yields of doped materials. QY_{total}: total photoluminescence quantum yields of the doped materials.

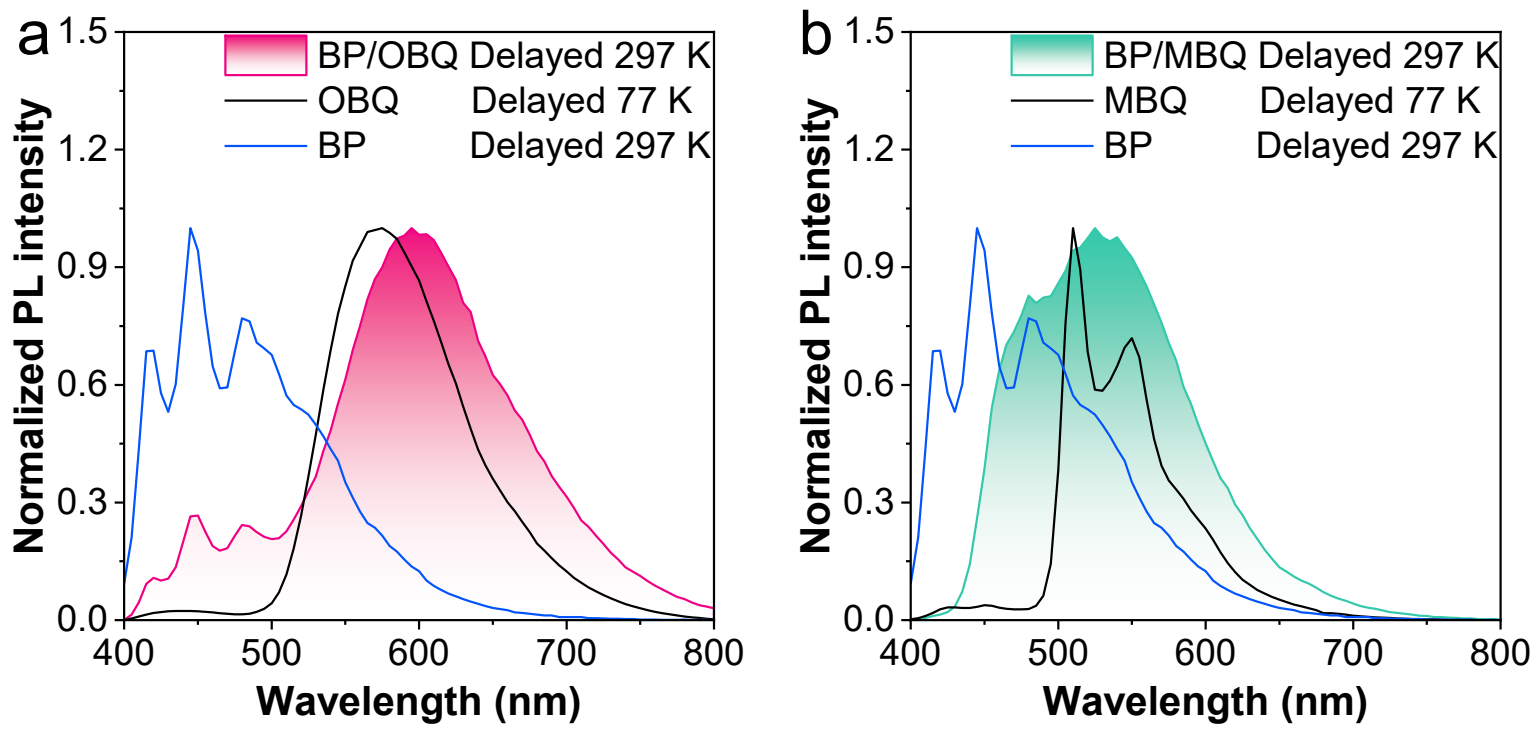

Figure S16. (a) Delayed emission spectra of BP, BP/OBQ solids at 297 K and OBQ in dilute solution (1.0×10^{-5} M in 2-methyltetrahydrofuran) at 77 K. (b) Delayed emission spectra of BP, BP/MBQ solids at 297 K and MBQ in dilute solution (1.0×10^{-5} M in 2-methyltetrahydrofuran) at 77 K. Excitation wavelength: λ_{ex}: 360 nm. Delay time 1 ms.

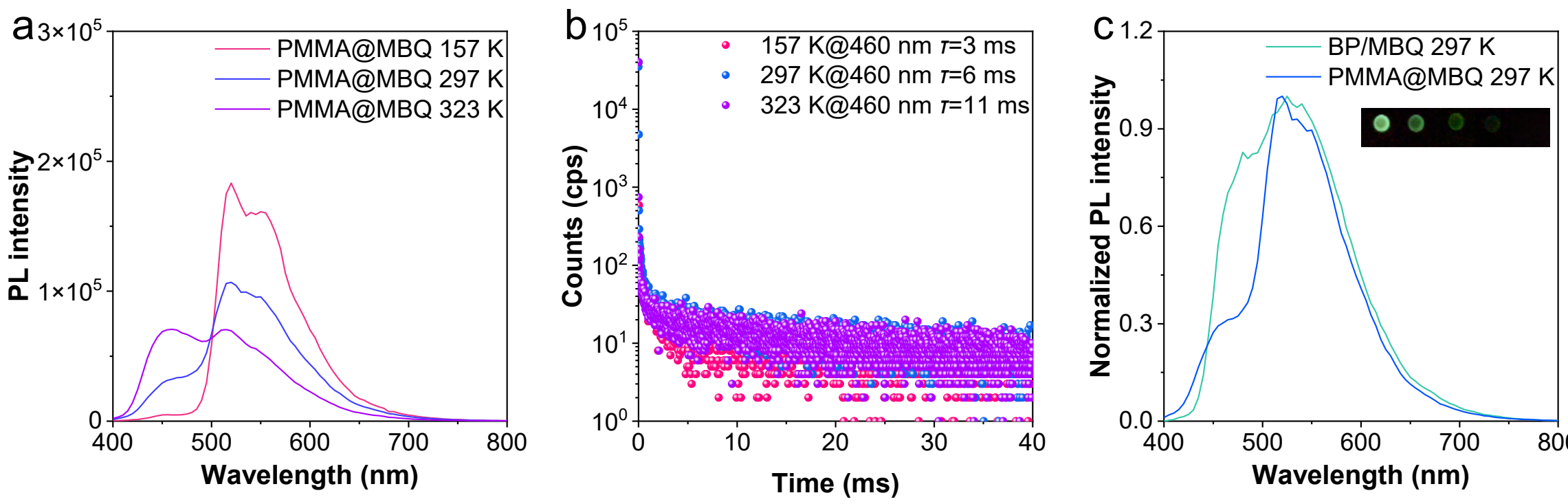

Figure S17. (a) Delayed emission spectra of PMMA@MBQ at different temperatures under vacuum. (b) Time-resolved emission decay curves of PMMA@MBQ at different temperatures under vacuum. (c) Normalized delayed emission spectra of BP/MBQ at the atmospheric environment and PMMA@MBQ under vacuum. Inset: PMMA@MBQ phosphorescence photograph at the atmospheric environment. Excitation wavelength: λ_{ex}: 360 nm. Delay time 1 ms.

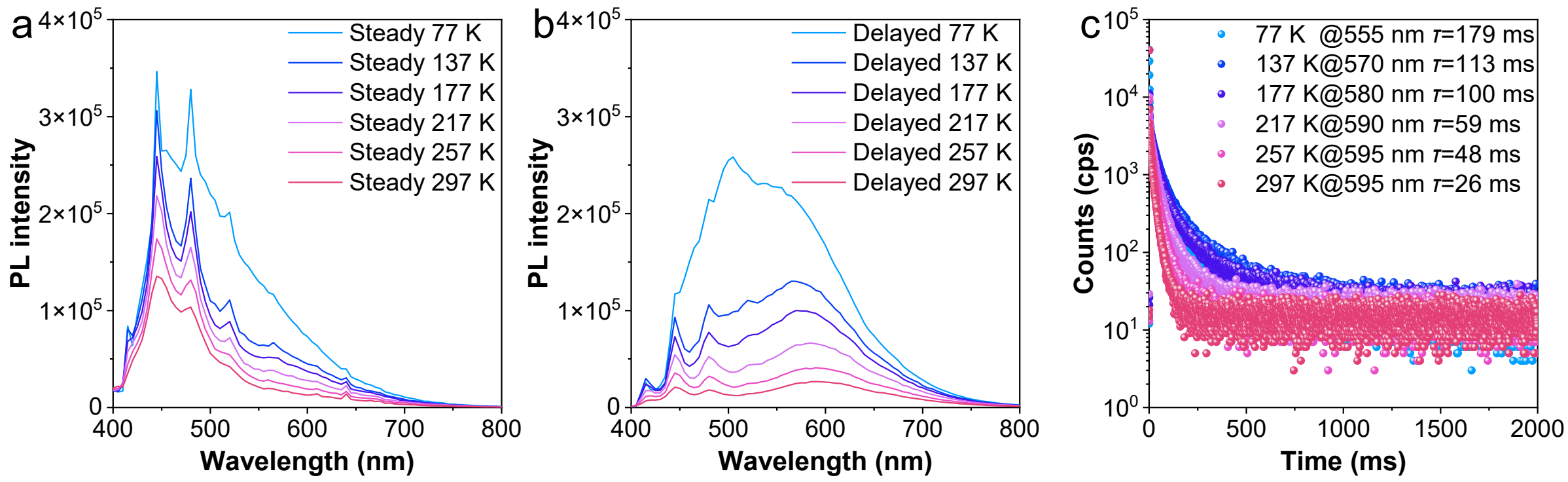

Figure S18. (a) The steady-state emission spectra, (b) delayed emission spectra and (c) time-resolved emission decay curves of BP/OBQ at different temperatures. Excitation wavelength: λ_{ex}: 360 nm. Delay time 1 ms.

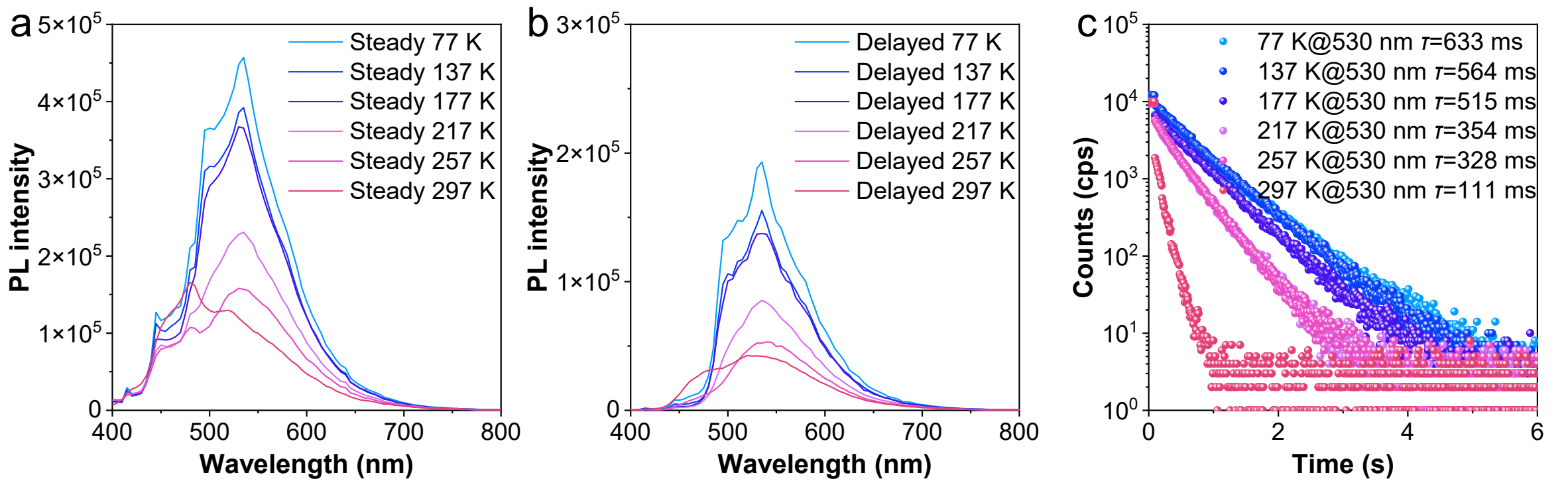

Figure S19. (a) The steady-state emission spectra, (b) delayed emission spectra and (c) time-resolved emission decay curves of BP/MBQ at different temperatures. Excitation wavelength: λ_{ex}: 360 nm. Delay time 1 ms.

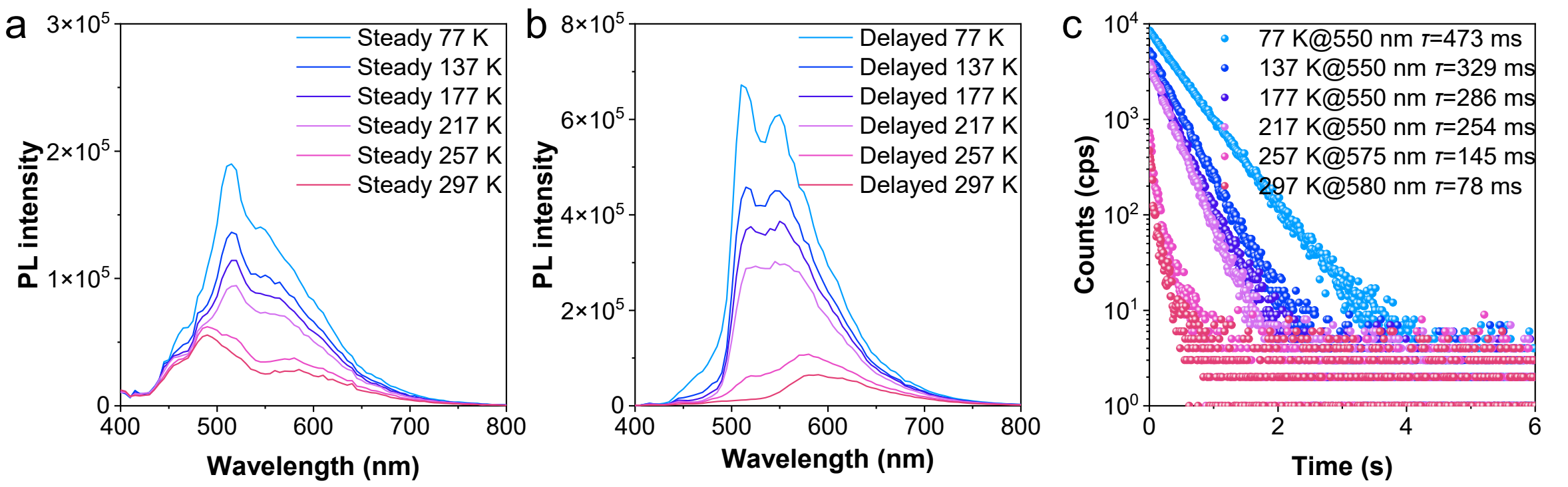

Figure S20. (a) The steady-state emission spectra, (b) delayed emission spectra and (c) time-resolved emission decay curves of BP/PBQ at different temperatures. Excitation wavelength: λ_{ex}: 360 nm. Delay time 1 ms.

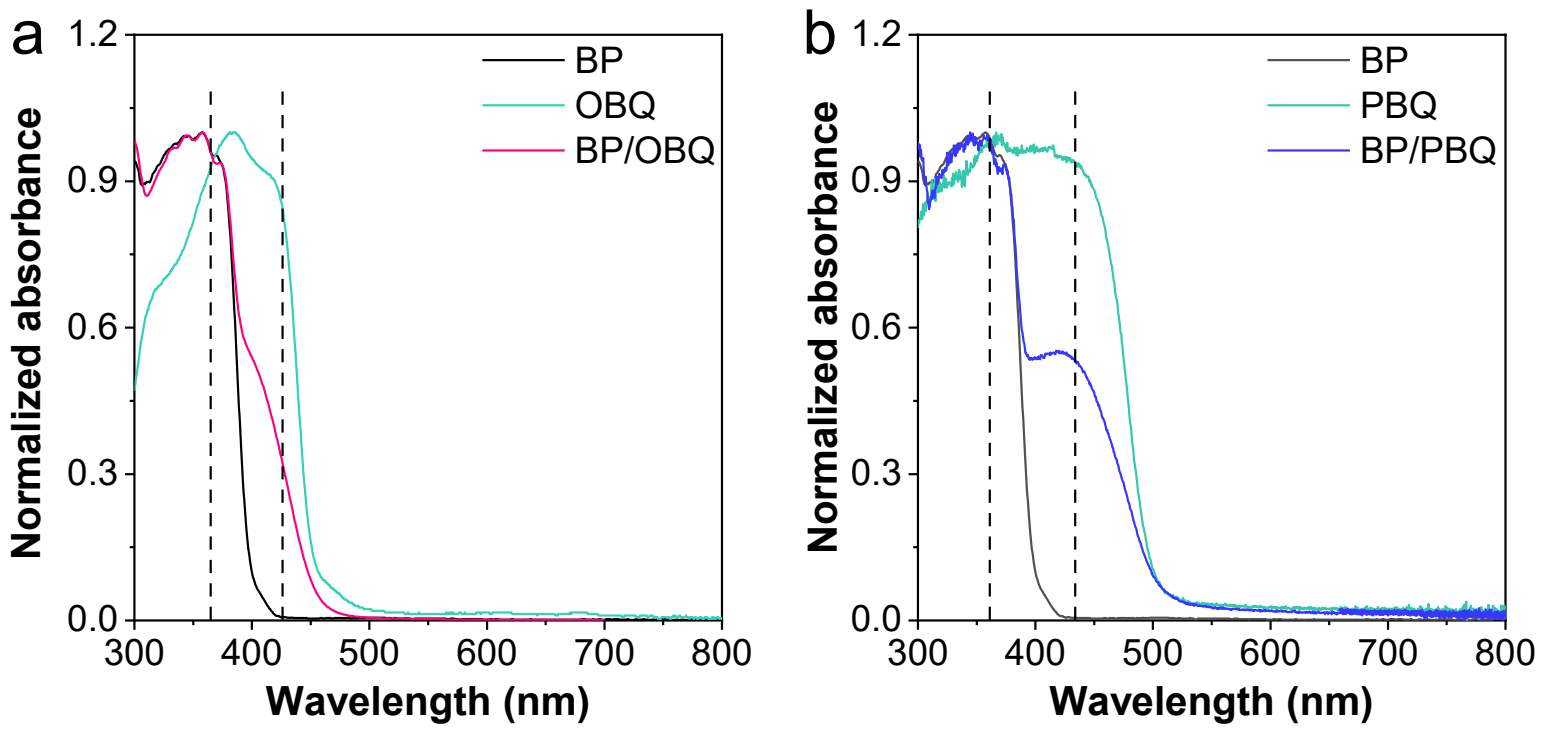

Figure S21. (a) Normalized UV-vis absorption spectra of solid BP, OBQ, and BP/OBQ. (b) Normalized UV-vis absorption spectra of solid BP, PBQ, and BP/PBQ. The dashed lines indicate the selected excitation wavelengths of 360 and 430 nm.

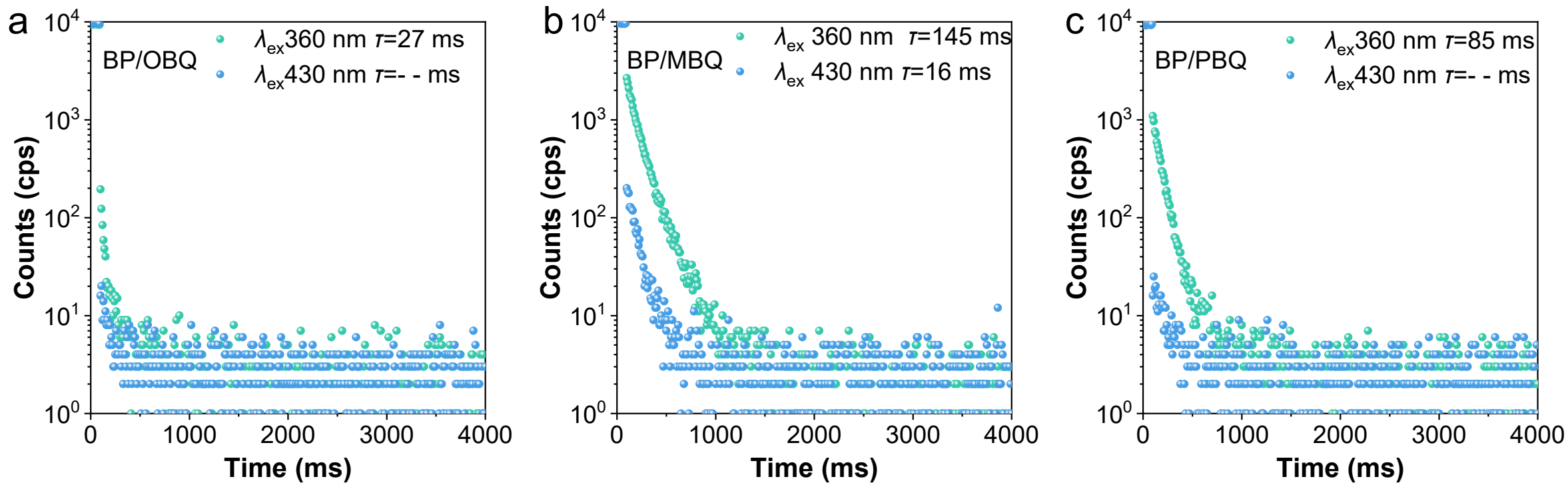

Figure S22. Time-resolved emission decay curves of BP/OBQ (a), BP/MBQ (b), and BP/PBQ (c) under excitation at 360 or 430 nm.

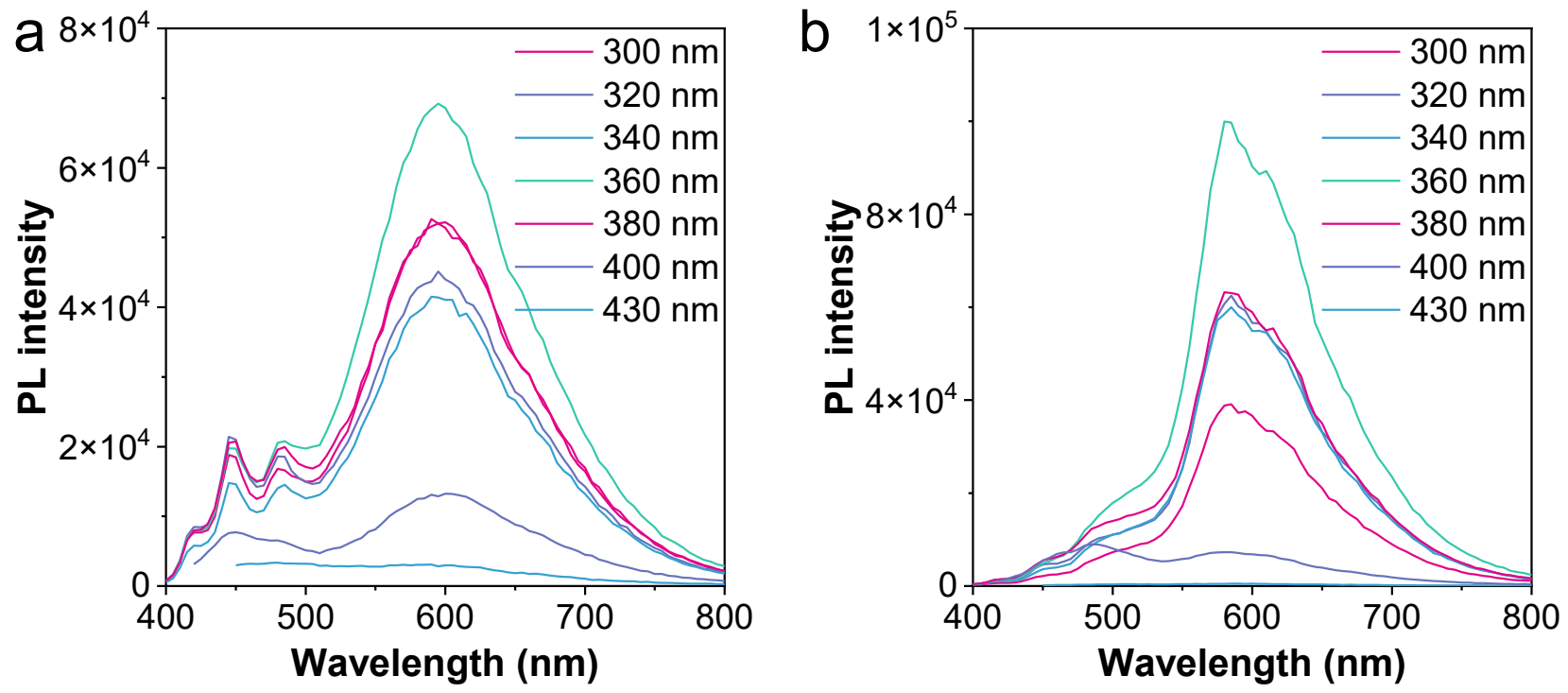

Figure S23. Delayed emission spectra of BP/OBQ (a) and BP/PBQ (b) under different excitation wavelengths.

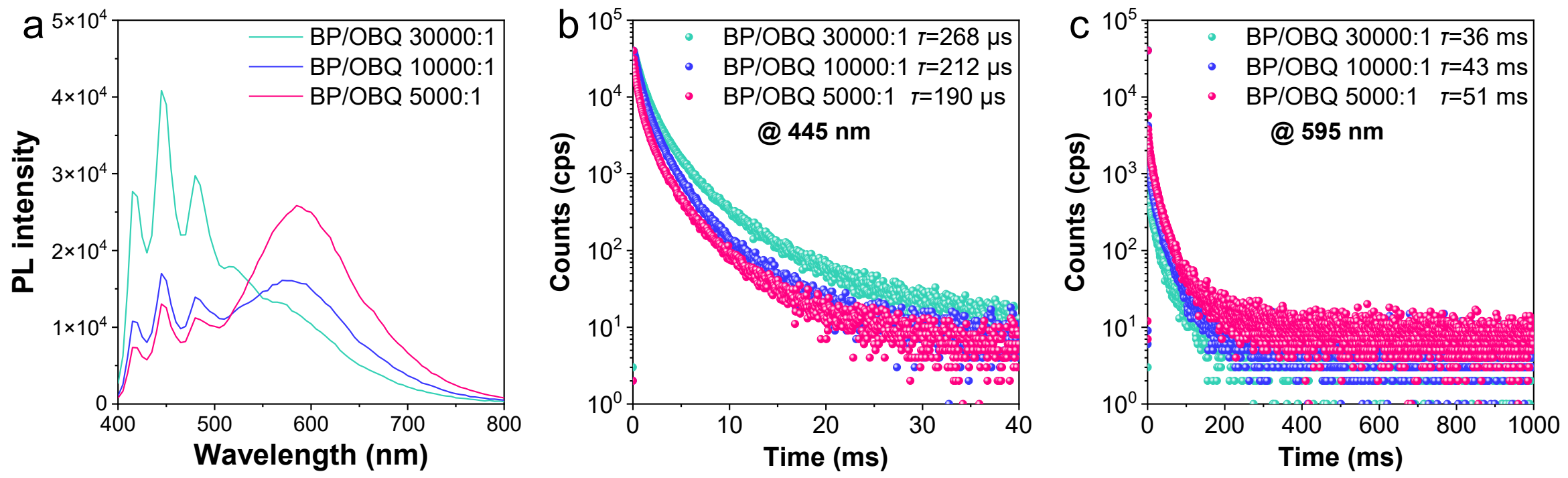

Figure S24. (a) Delayed emission spectra of BP/OBQ at different mass ratios. Time-resolved emission decay curves of BP/OBQ at 445 nm (b) and 595 nm (c) at different doped mass ratios. Excitation wavelength: λ_{ex}: 360 nm. Delay time 1 ms.

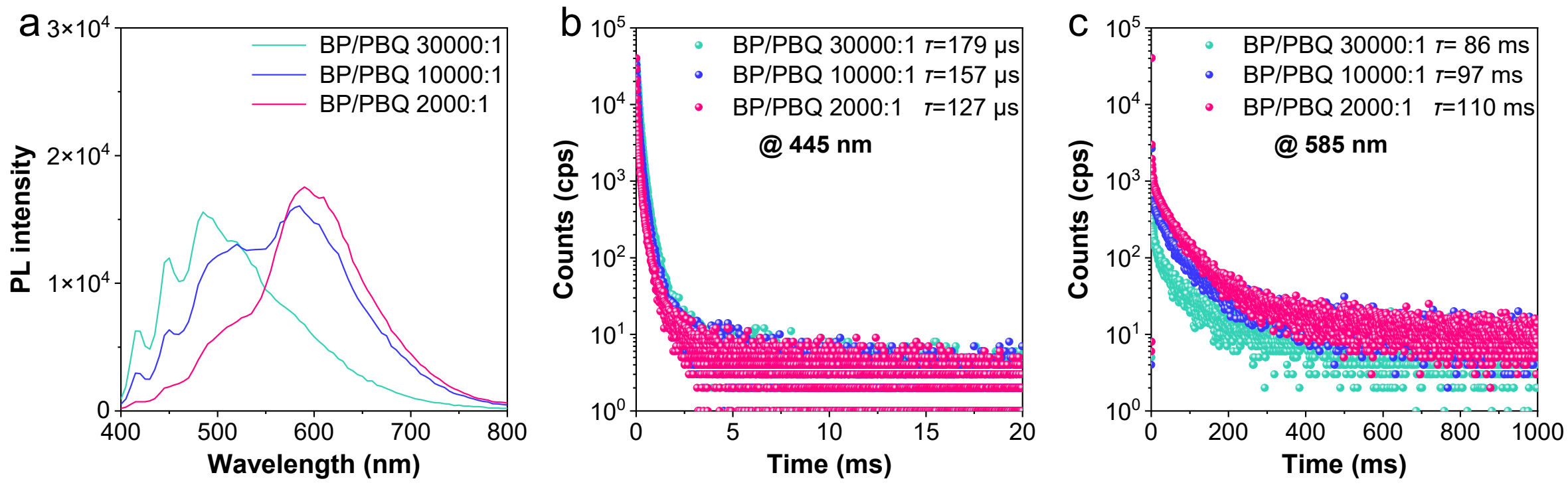

Figure S25. (a) Delayed emission spectra of BP/PBQ at different mass ratios. Decay curves of BP/PBQ at 445 nm (b) and 585 nm (c) at different doping mass ratios. Excitation wavelength: λ_{ex}: 360 nm. Delay time 1 ms.

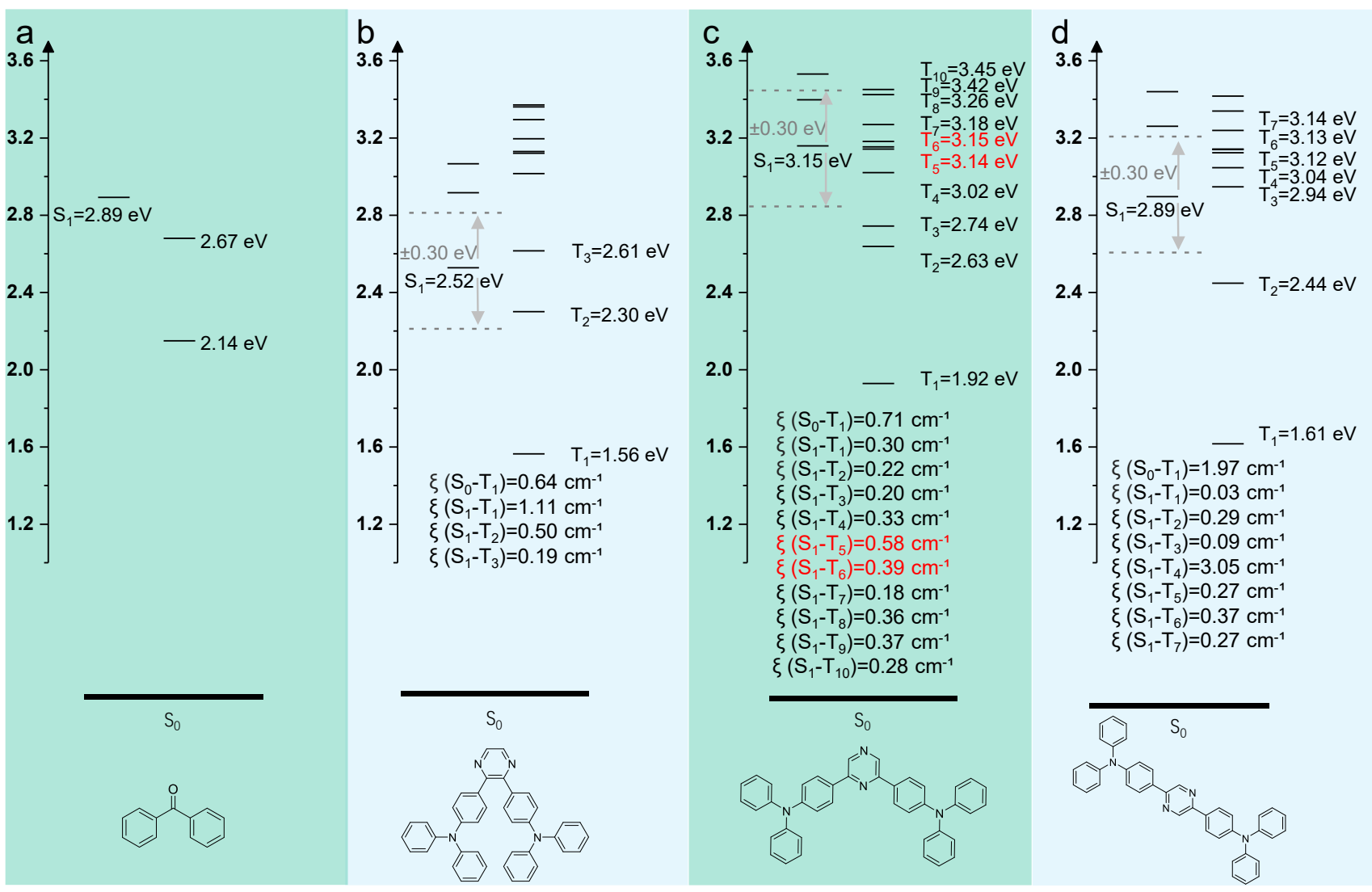

Figure S26. Diagrams of the TD-DFT calculated energy levels and SOC constants of BP (a), OBQ (b), MBQ (c), and PBQ (d). SOC matrix elements of S_1-T_n transitions available for ISC processes were highlighted in red. ξ represents the SOC matrix element. (The geometries were extracted from single crystals).

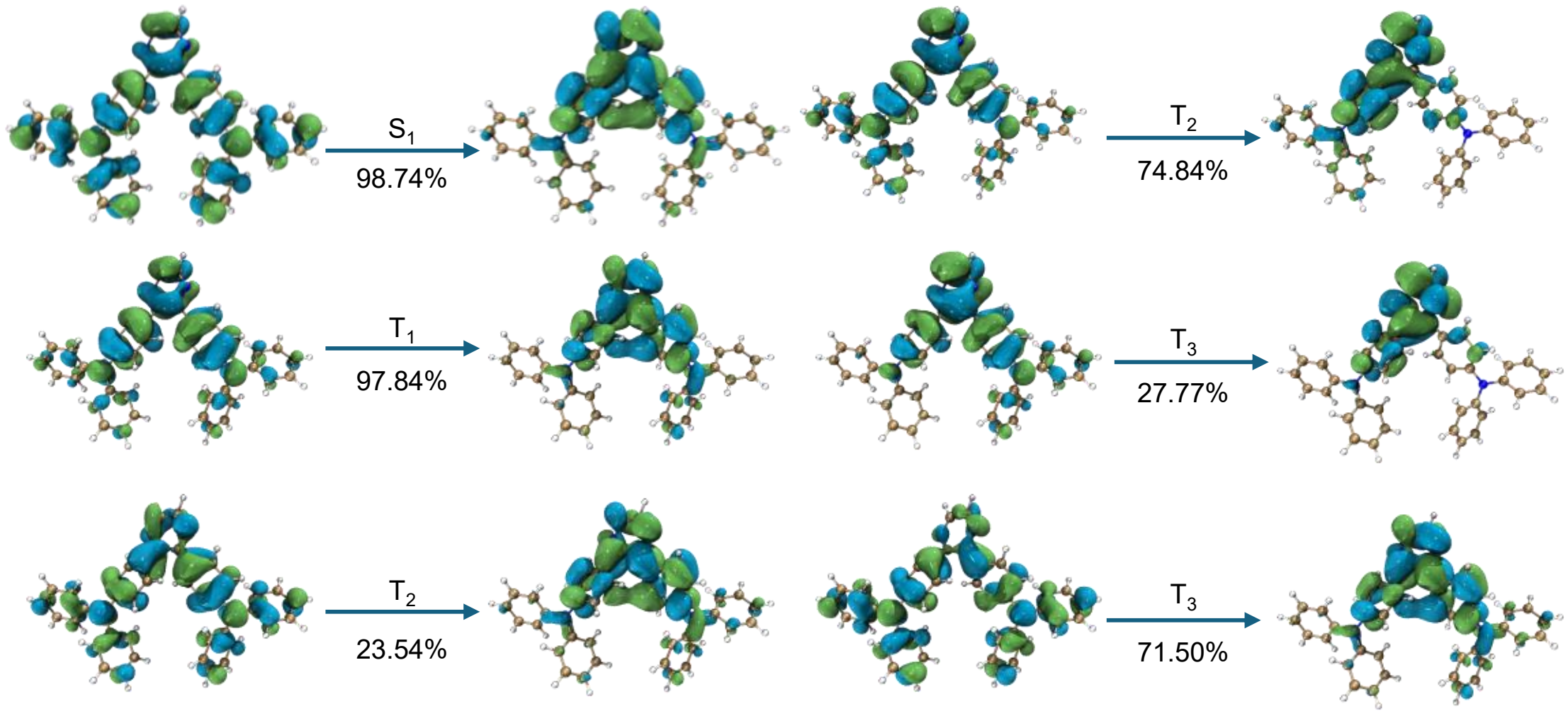

Figure S27. The NTO analyses of the S_1, T_1, T_2, and T_3 states of OBQ.

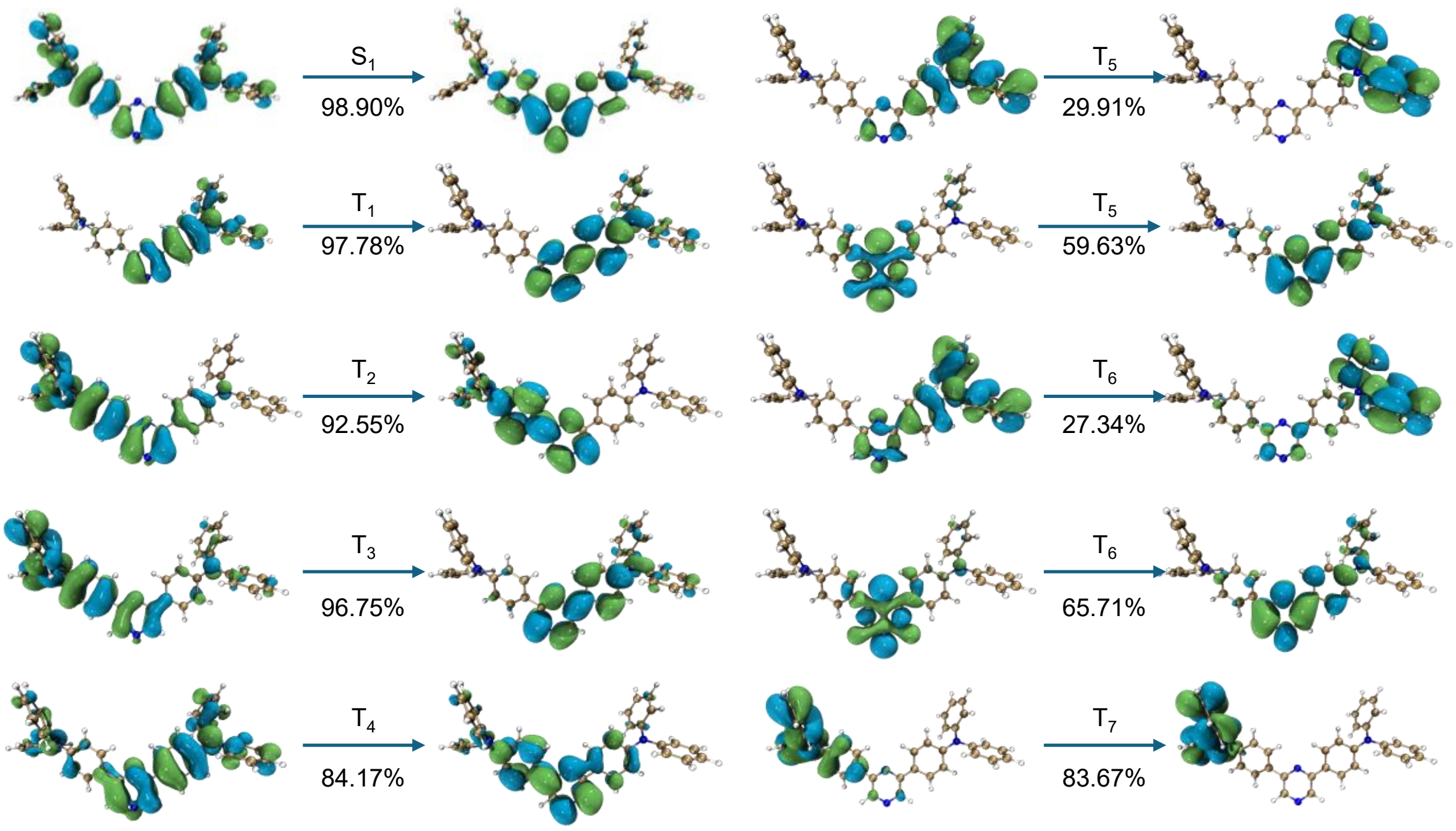

Figure S28. The NTO analyses of the S_1, T_1, T_2, T_3, T_4, T_5, T_6, and T_7 states of MBQ.

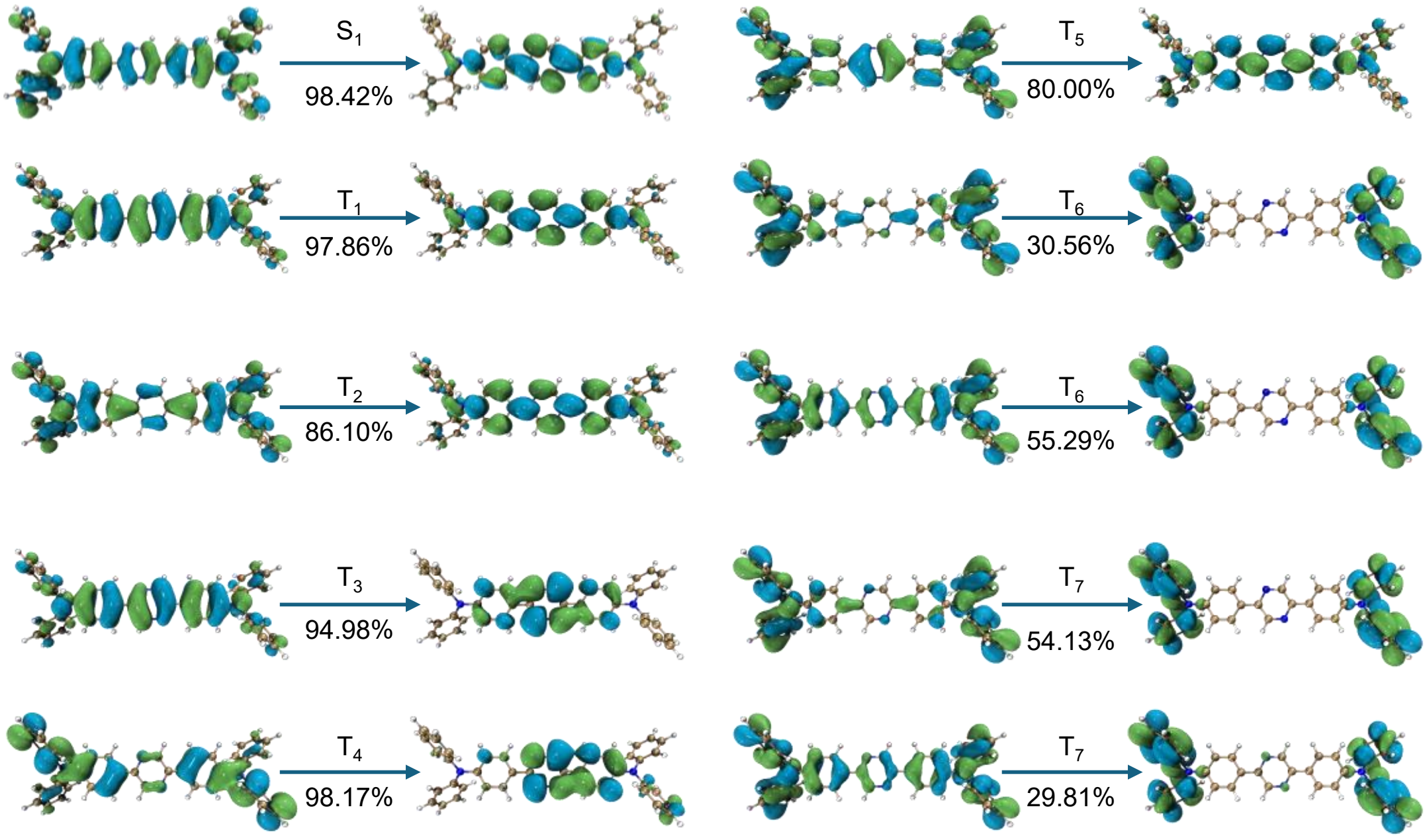

Figure S29. The NTO analyses of the S_1, T_1, T_2, T_3, T_4, T_5, T_6, and T_7 states of PBQ.

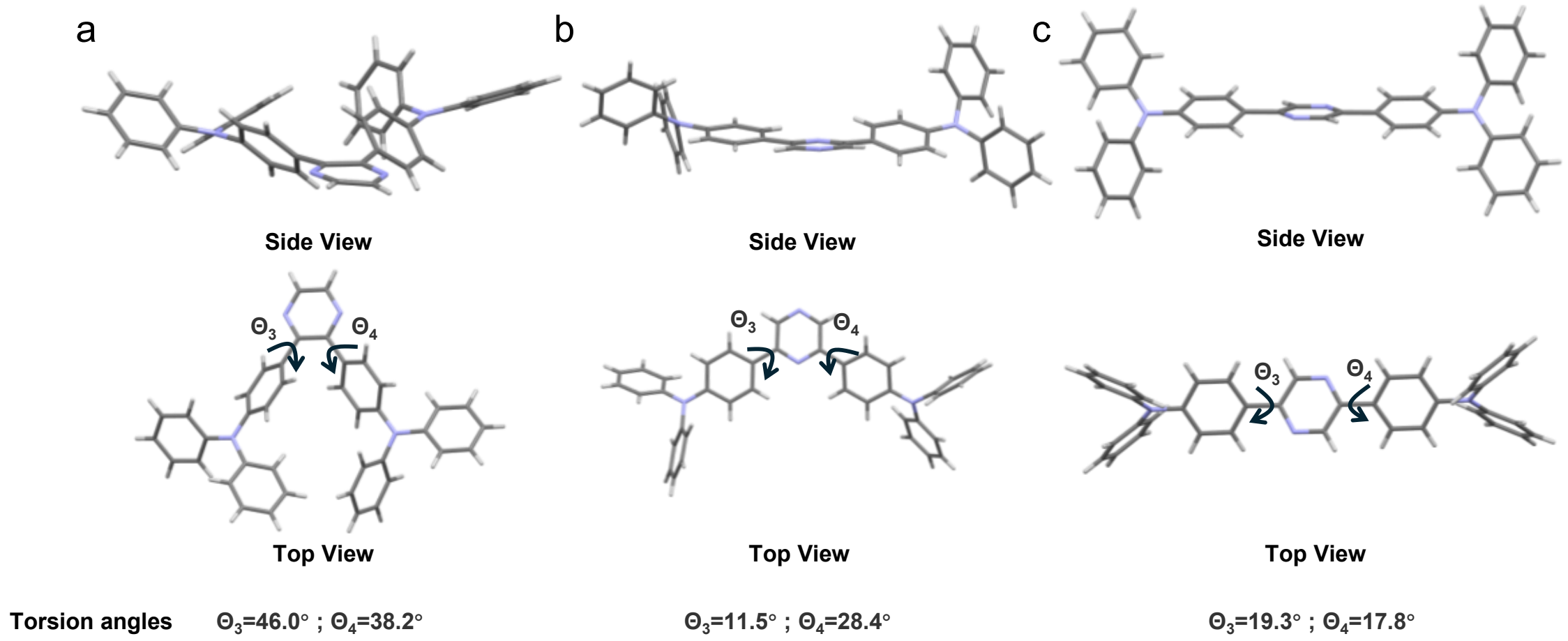

Figure S30. Molecular conformations of OBQ (a), MBQ (b), and PBQ (c) extracted from the corresponding single crystal structures from different views. The selected torsion angles are indicated.

Table S2. Crystal data and structure refinement for OBQ, MBQ, PBQ.

Name	OBQ	MBQ	PBQ
Empirical formula	$C_{40}H_{30}N_4$	$C_{40}H_{30}N_4$	$C_{40}H_{30}N_4$
Formula weight	566.68	566.68	566.68
Temperature/K	150.05	293.15	149.99
Crystal system	monoclinic	monoclinic	monoclinic
Space group	$P2_1/n$	$P2_1/n$	$P2_1/n$
a (Å)	15.9620(2)	10.89179(17)	6.9300(2)
b (Å)	12.61180(10)	17.1106(3)	14.5406(4)
c (Å)	16.6172(2)	16.7102(3)	15.0958(4)
α (°)	90	90	90
β (°)	116.252(2)	105.2140(16)	98.091(2)
γ (°)	90	90	90
Volume ($Å^3$)	3000.17(7)	3005.04(8)	1506.01(7)
Z	4	4	2
Density (g/cm^3)	1.255	1.253	1.250
F(000)	1192.0	1192.0	596.0
Crystal size/mm^3	0.16 × 0.14 × 0.12	0.15 × 0.12 × 0.11	0.14 × 0.12 × 0.1
CCDC Number	2545022	2545024	2545026

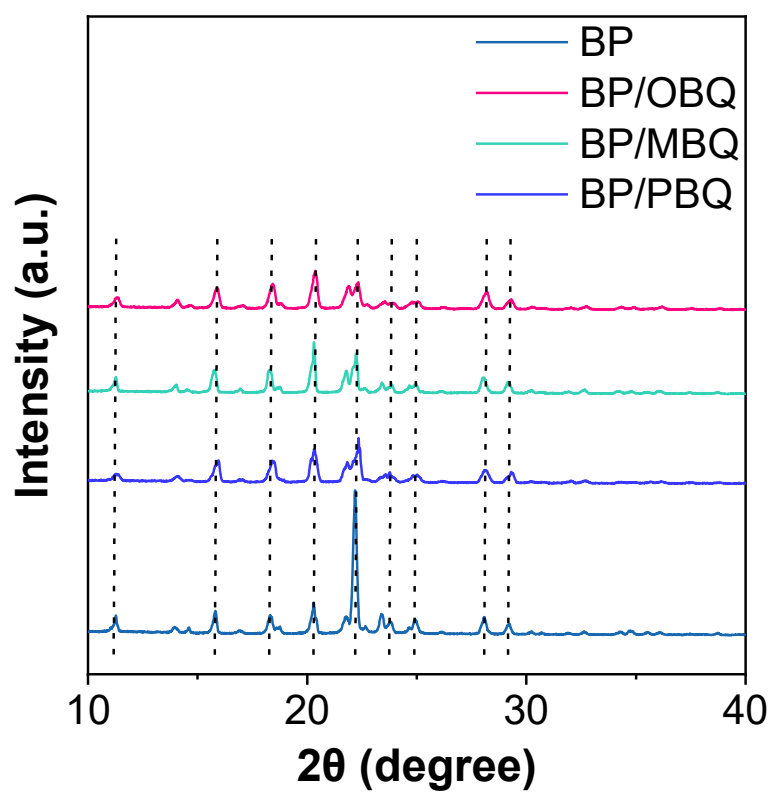

Figure S31. The PXRD patterns of pristine BP, BP/OBQ, BP/MBQ, and BP/PBQ doped materials.

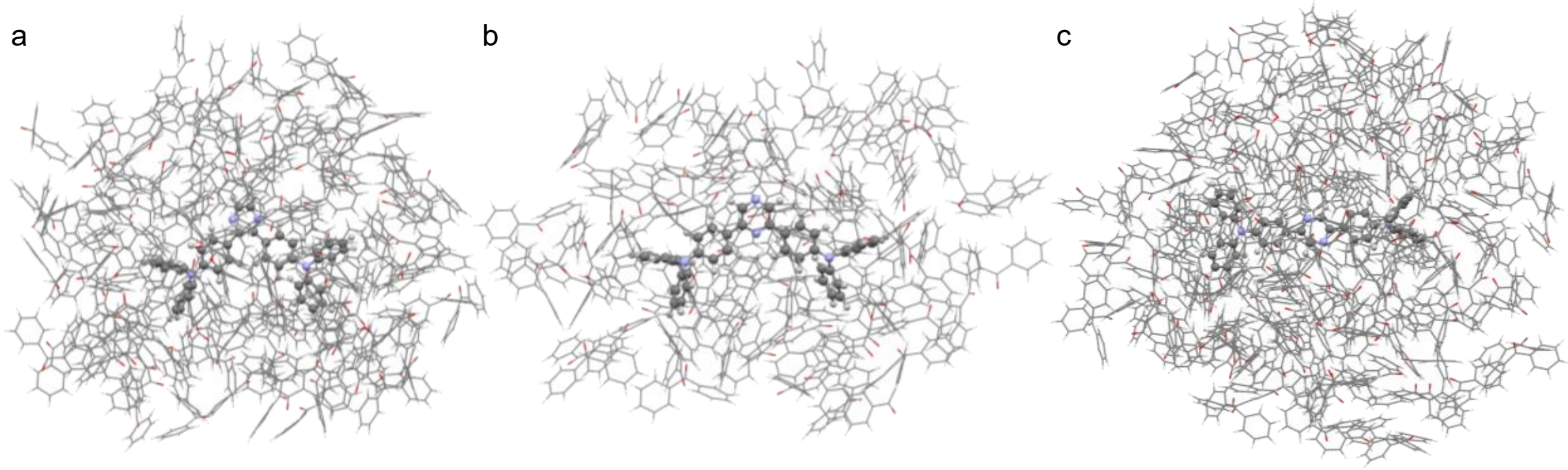

Figure S32. QM/MM model setup of BP/OBQ (a), BP/MBQ (b), and BP/PBQ (c) doped systems.

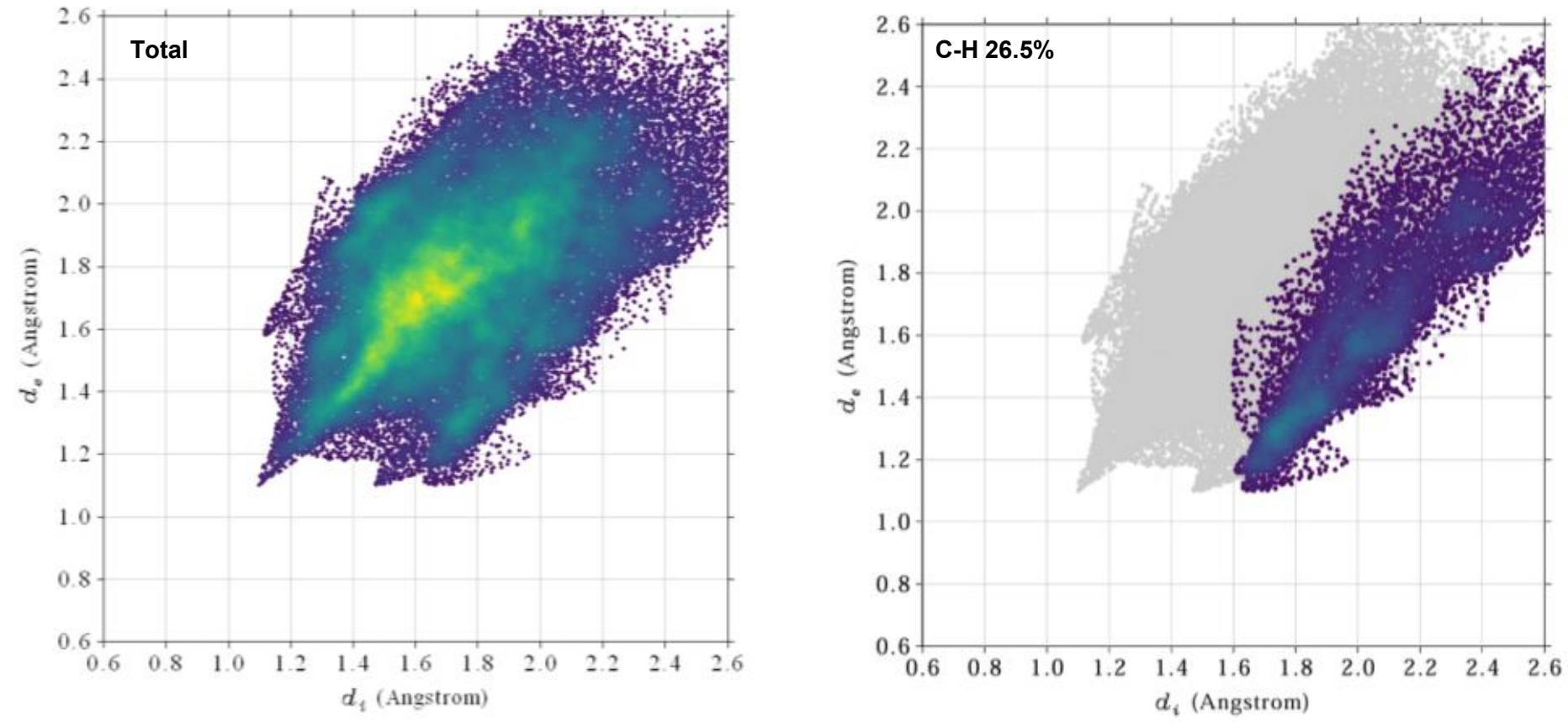

Figure S33. Two-dimensional fingerprint plots analysis of BP/OBQ calculated from the Hirshfeld surface, including all contacts (left) and the individual C-H (right) intermolecular interactions.

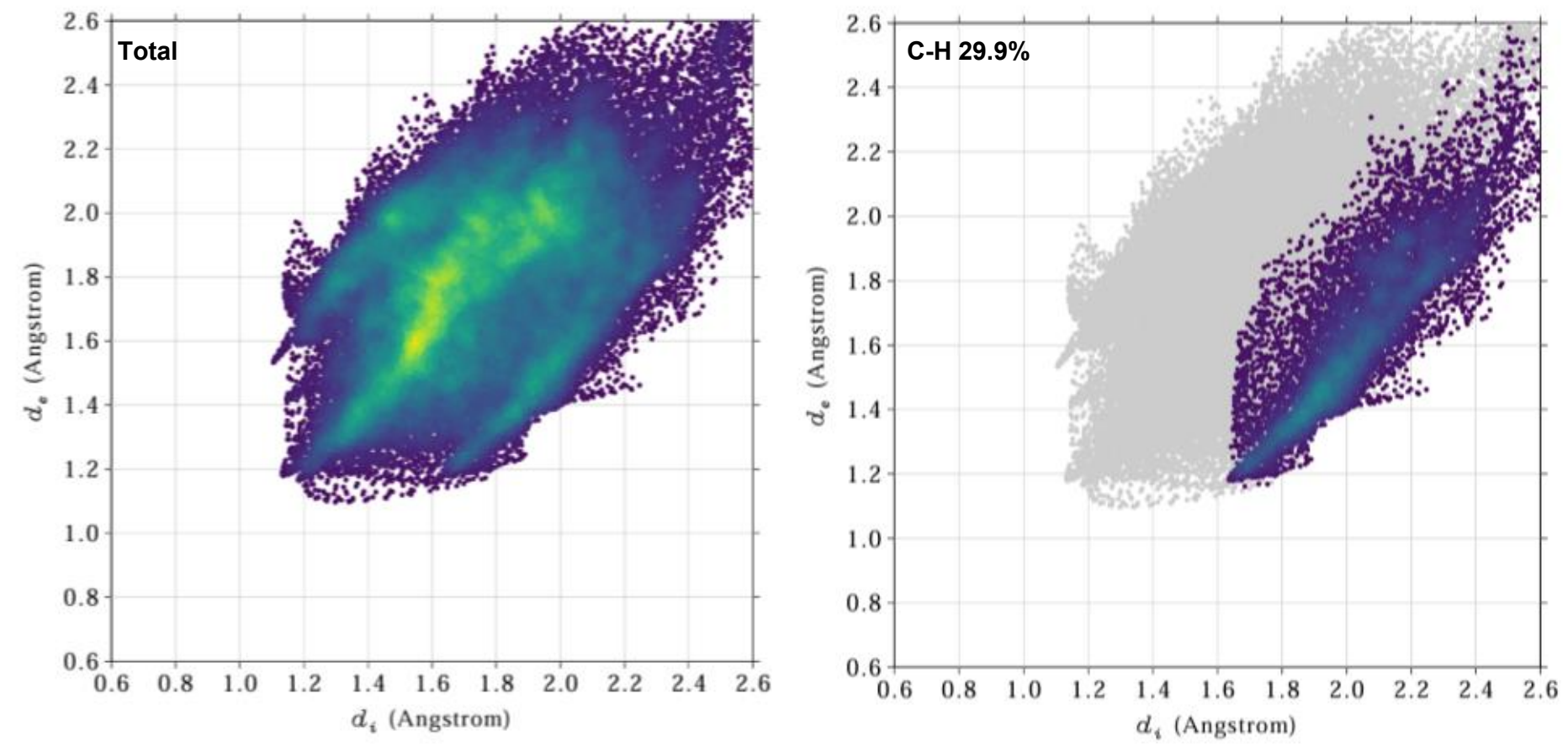

Figure S34. Two-dimensional fingerprint plots analysis of BP/MBQ calculated from the Hirshfeld surface, including all contacts (left) and the individual C-H (right) intermolecular interactions.

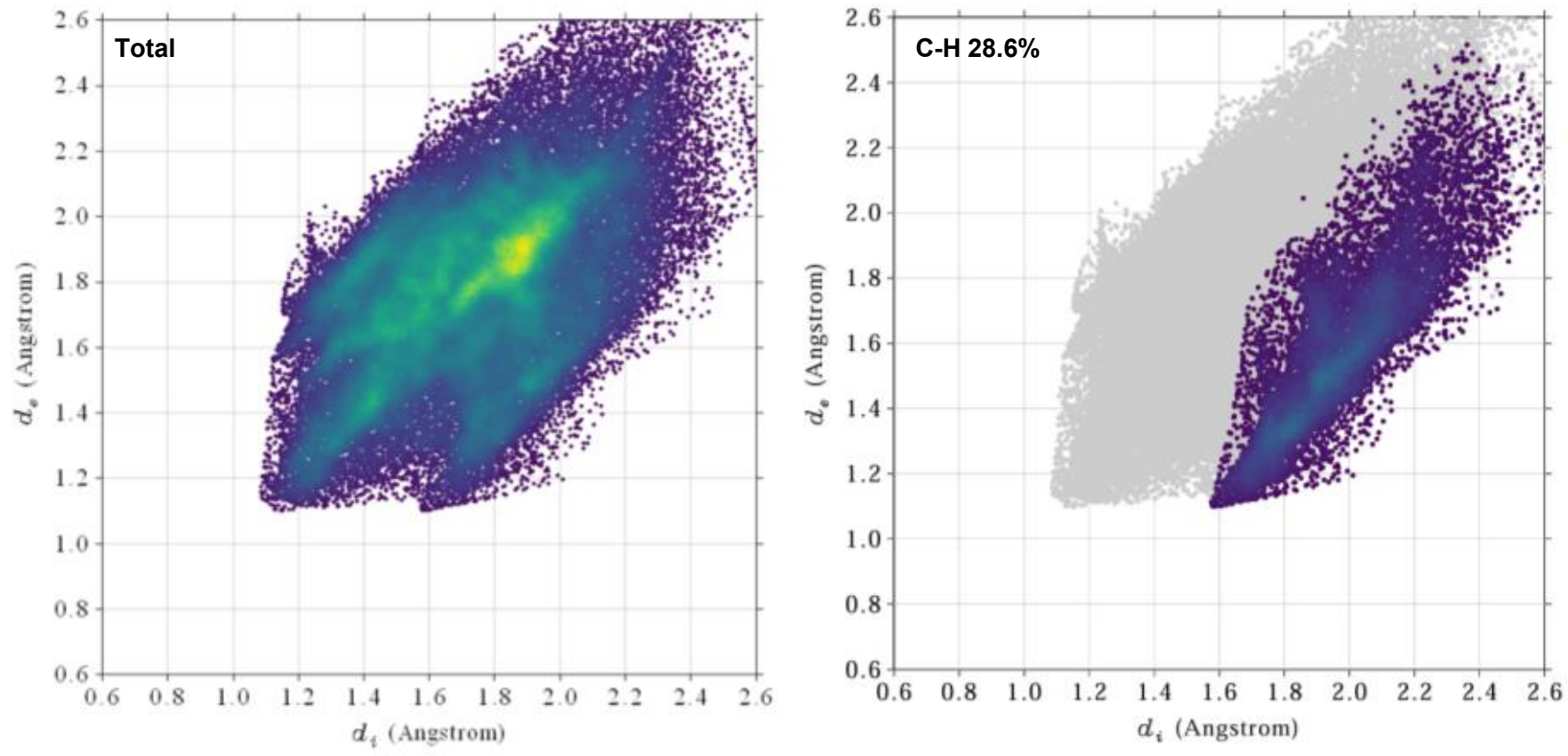

Figure S35. Two-dimensional fingerprint plots analysis of BP/PBQ calculated from the Hirshfeld surface, including all contacts (left) and the individual C-H (right) intermolecular interactions.

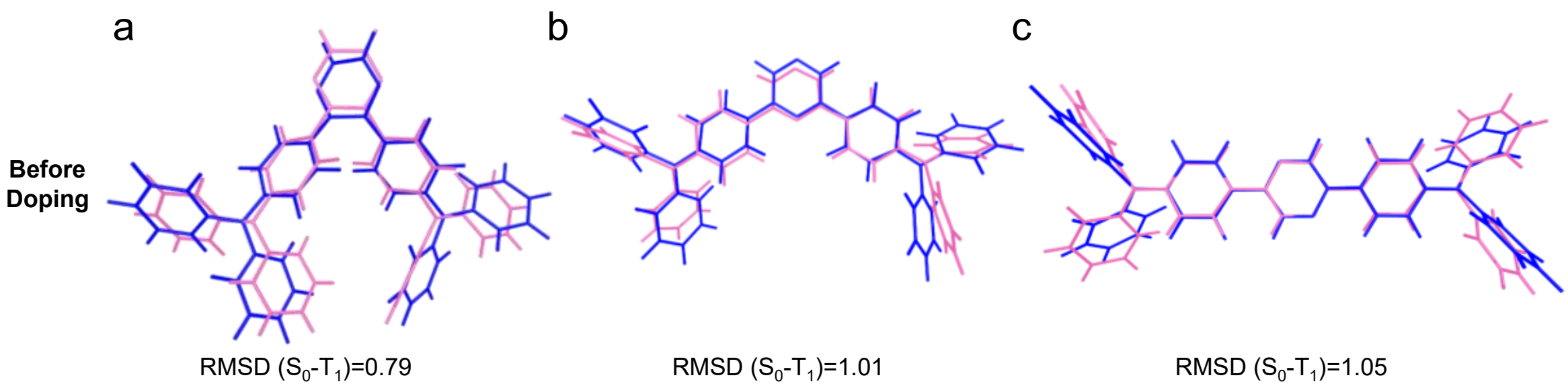

Figure S36. Overlaid molecular geometries of OBQ (a), MBQ (b), and PBQ (c) before doping into BP, shown for the S_0 (blue) and T_1 (pink) states.

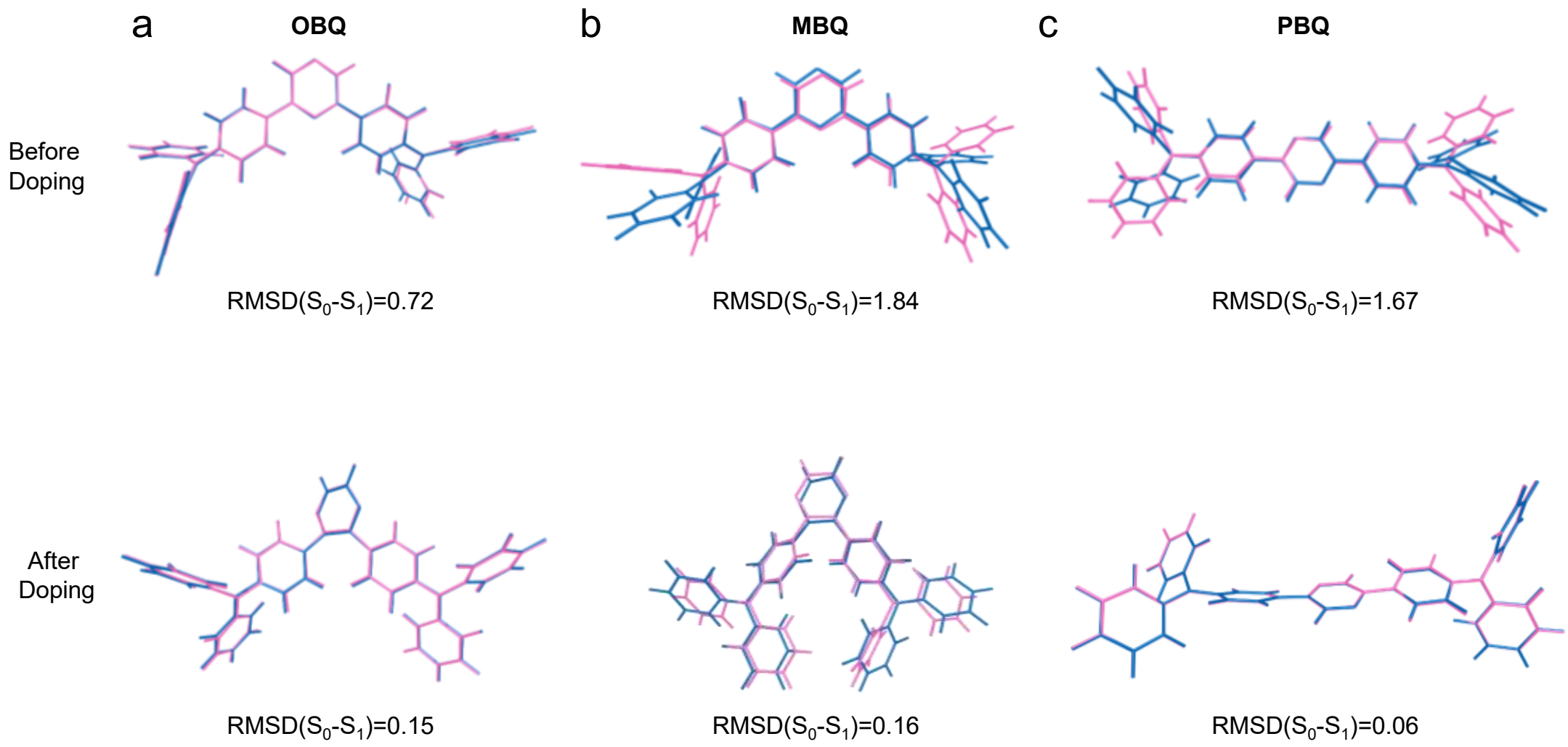

Figure S37. Overlaid molecular geometries of OBQ (a), MBQ (b), and PBQ (c) before and after doping into BP, shown for the S_0 (blue) and S_1 (pink) states, for RMSD comparison.

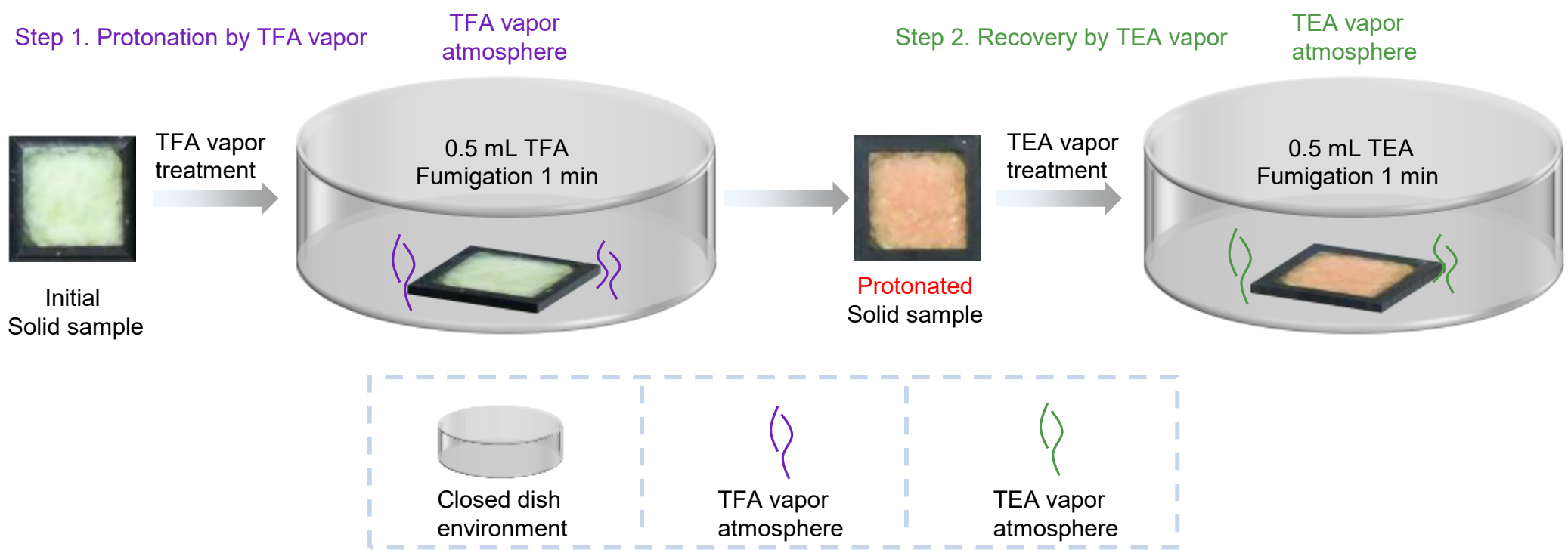

Figure S38. Schematic illustration of TFA vapor protonation and TEA vapor recovery treatment of all compounds.

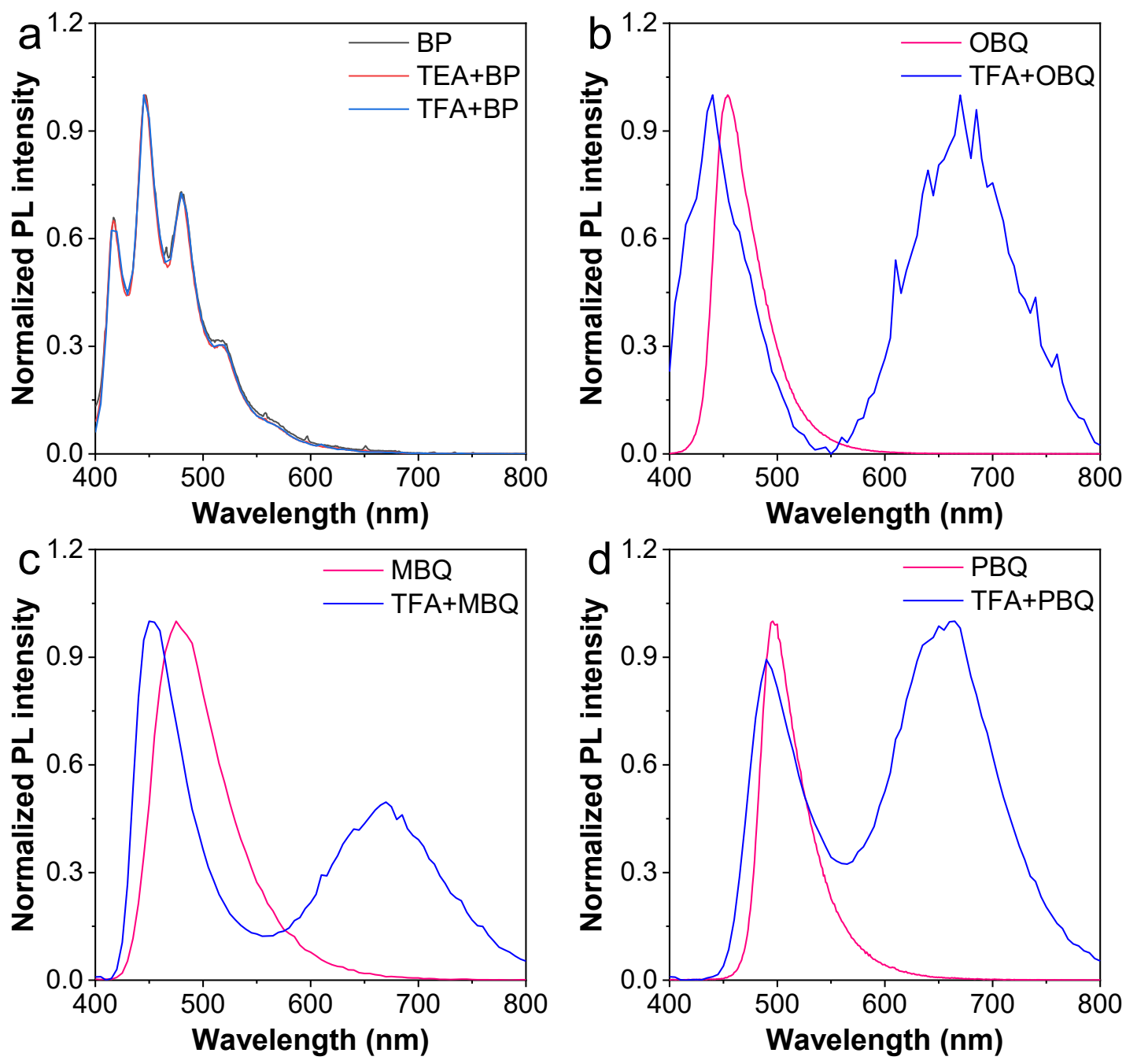

Figure S39. (a) Normalized steady-state emission spectra of BP powders in its pristine state, after protonation by TFA, and being fumed with TEA. Normalized steady-state emission spectra of OBQ (b), MBQ (c), and PBQ (d) powders before and after response to TFA stimulation.

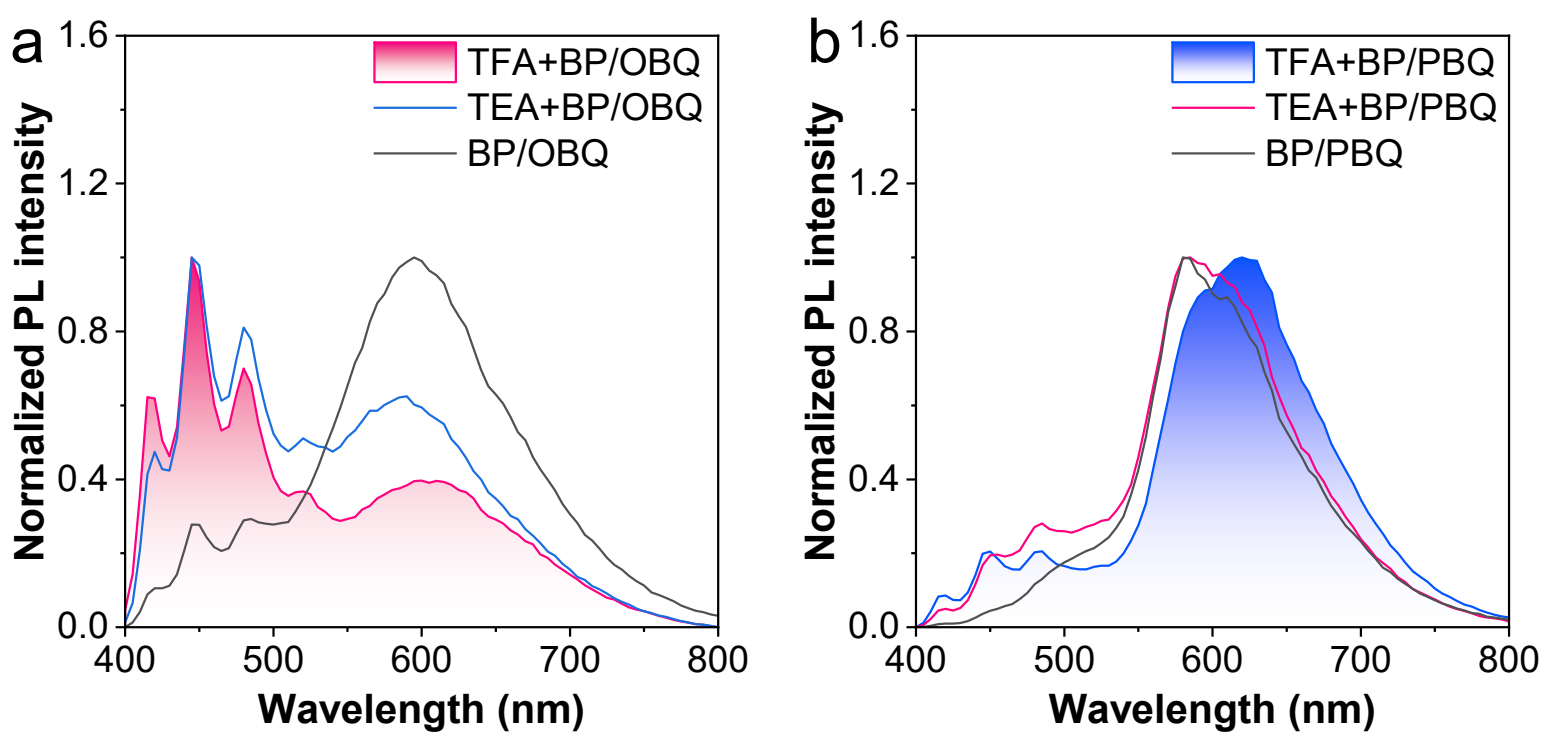

Figure S40. Normalized delayed emission spectra of BP/OBQ (a) and BP/PBQ (b) in the initial state and after TFA/TEA treatment (delay time 1 ms).

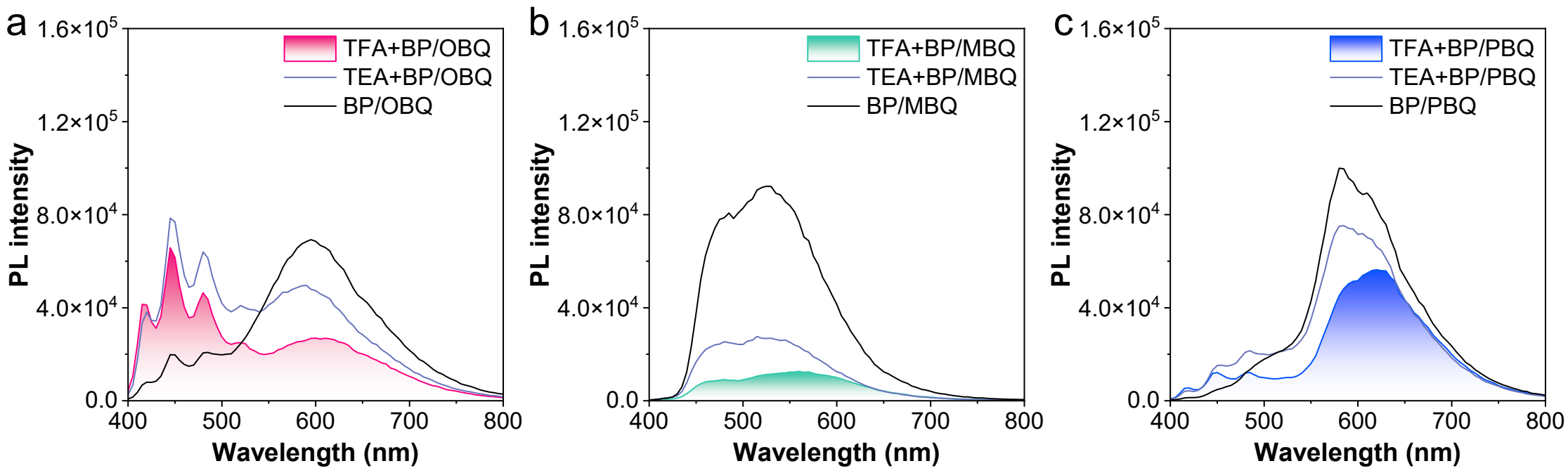

Figure S41. Delayed emission spectra of BP/OBQ (a), BP/MBQ (b), and BP/PBQ (c) in the initial state and after TFA/TEA treatment (delay time 1 ms).

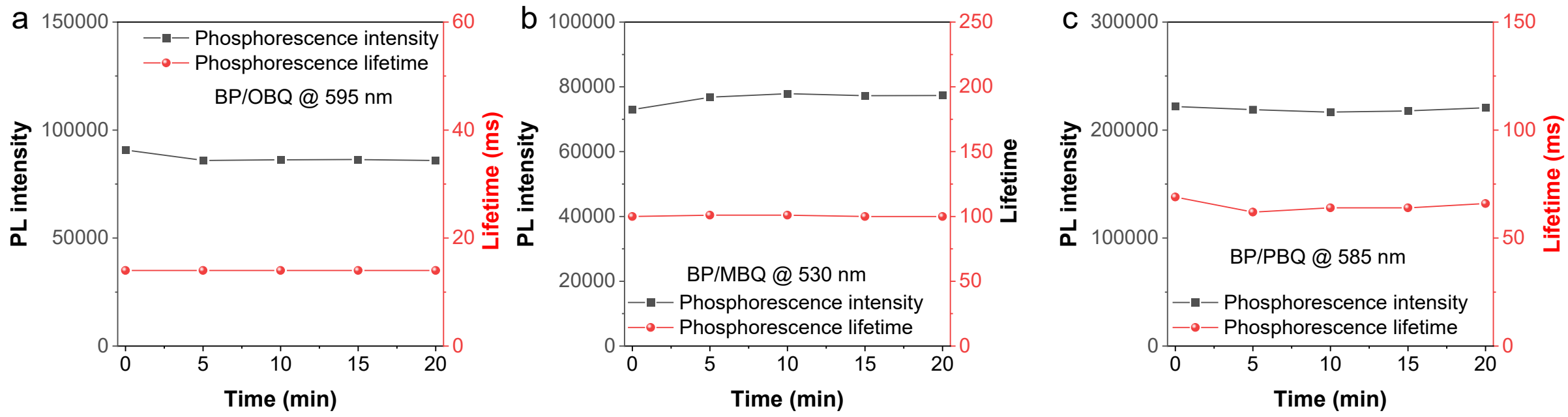

Figure S42. Phosphorescence intensities and lifetimes of BP/OBQ (a), BP/MBQ (b), and BP/PBQ (c) after thermal treatment at 310 K for different periods from 0 to 20 min. The gray squares and red circles represent the phosphorescence intensity and lifetime, respectively.

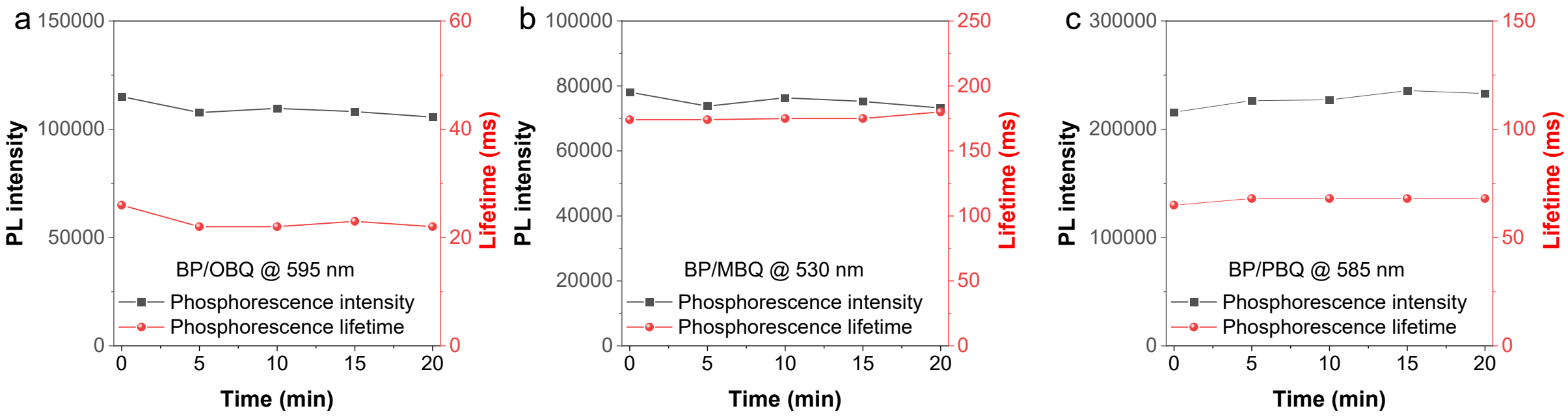

Figure S43. Phosphorescence intensities and lifetimes of BP/OBQ (a), BP/MBQ (b), and BP/PBQ (c) following continuous irradiation at 365 nm with a power density of 80 mW cm^{-2} for different periods from 0 to 20 min. The gray squares and red circles represent the phosphorescence intensity and lifetime, respectively.

Notes and References

1 M. J. Frisch, G. W. Trucks, H. B. Schlegel, G. E. Scuseria, M. A. Robb, J. R. Cheeseman, G. Scalmani, V. Barone, B. Mennucci, G. A. Petersson, H. Nakatsuji, M. Caricato, X. Li, H. P. Hratchian, A. F. Izmaylov, J. Bloino, G. Zheng, J. L. Sonnenberg, M. Hada, M. Ehara, K. Toyota, R. Fukuda, J. Hasegawa, M. Ishida, T. Nakajima, Y. Honda, O. Kitao, H. Nakai, T. Vreven, J. A. Montgomery, Jr., J. E. Peralta, F. Ogliaro, M. Bearpark, J. J. Heyd, E. Brothers, K. N. Kudin, V. N. Staroverov, R. Kobayashi, J. Normand, K. Raghavachari, A. Rendell, J. C. Burant, S. S. Iyengar, J. Tomasi, M. Cossi, N. Rega, J. M. Millam, M. Klene, J. E. Knox, J. B. Cross, V. Bakken, C. Adamo, J. Jaramillo, R. Gomperts, R. E. Stratmann, O. Yazyev, A. J. Austin, R. Cammi, C. Pomelli, J. W. Ochterski, R. L. Martin, K. Morokuma, V. G. Zakrzewski, G. A. Voth, P. Salvador, J. J. Dannenberg, S. Dapprich, A. D. Daniels, Ö. Farkas, J. B. Foresman, J. V. Ortiz, J. Cioslowski, and D. J. Fox, *Gaussian 09*. Gaussian, Inc.: Wallingford, CT, USA, 2009.

2 F. Neese, *WIREs Comput. Mol. Sci.*, 2022, **12**, e1606.

3 T. Lu and F. Chen, *J. Comput. Chem.*, 2012, **33**, 580-592.

4 W. Humphrey, A. Dalke and K. Schulten, *J. Mol. Graph.*, 1996, **14**, 33-38.

5 L. Jin, Z. Wang, W. Mo, H. Deng, W. Hong and Z. Chi, *Angew. Chem. Int. Ed.*, 2024, **63**, e202410974.
